# High-Throughput Amplicon Sequencing Microbial Communities and Volatile Flavour Compounds of Naturally Air-Dried Yak Jerky from Two Regions of Xizang

**DOI:** 10.3390/microorganisms14092058

**Published:** 2026-09-15

**Authors:** Jinchao Xu, Xiaolin Cai, Cuiting Liu, Guipeng Li, Liwei Zang, Keke Li, Zhendong Liu, Yifan Zhang

**Affiliations:** 1 Key Laboratory of Deep Processing and High-Value Utilization of Characteristic Agricultural and Livestock Products of Xizang Autonomous Region, Food Science College, Xizang Agriculture & Animal Husbandry University, Nyingchi 860000, China; 13684565008@163.com (J.X.); 13647768607@163.com (X.C.); cc17771954378@163.com (C.L.); 17393197853@126.com (G.L.); 18866353246@163.com (L.Z.); 18712507959@163.com (K.L.); 2 Institute of Agricultural Product Quality Standard and Testing Research, Xizang Academy of Agricultural and Animal Husbandry Sciences, Lhasa 850000, China

**Keywords:** high-throughput sequencing, fermented meat, Tibetan food, microflora, volatile flavour substances

## Abstract

Nagqu and Nyingchi, two typical regions in Xizang with distinctly contrasting climatic conditions, are highly representative of the complex and diverse climate patterns across the plateau. This study compared the microbial community structures and volatile compound profiles of naturally air-dried yak jerky produced in these two areas. Significant differences in key physicochemical properties, including pH, moisture content, color, and texture, were also detected between the jerky samples from the two regions. Using high-throughput sequencing and headspace solid phase microextraction-gas chromatograph-mass spectrometry, results revealed significant regional variations in the microbial communities and volatile organic compound (VOC) of yak jerky. For Nagqu City, the dominant bacterial and fungal genera were *Gemmiger* and *Pseudogymnoascus*, respectively; whereas in Nyingchi, the dominant bacterial and fungal genera were *Brochothrix* and *Naganishia*, respectively. A total of 94 VOCs were identified in the yak jerky, categorized into six classes: 18 aldehydes, 37 esters, 8 alcohols, 3 ketones, 12 hydrocarbons, and 16 others. Esters were identified as the potential contributors to the characteristic aroma profile of yak jerky. These findings provide valuable insights for the standardization and industrial production of yak jerky in Xizang. As this work analysed only four air-dried samples, it should be regarded as a preliminary characterisation; the number of samples needs to be enlarged in further studies.

## 1. Introduction

Yaks inhabit high-altitude, hypoxic, and cold environments. Their meat is rich in high-quality proteins, minerals, and unsaturated fatty acids, with a fat content significantly lower than that of conventional beef, making it well-suited for modern healthy diets [1,2]. As the most representative processed yak meat product, yak jerky has a long history and unique food cultural value. It is produced by open-air natural dehydration without starter-culture inoculation or strictly controlled fermentation conditions. Nevertheless, spontaneous microbial succession occurs on meat surfaces during the natural air-drying process, and for this reason, it is often regarded as a spontaneously fermented-like meat product in local folk processing. Traditional air-dried yak meat in the Xizang Autonomous Region relies on natural air-drying, facilitated by low temperatures, intense ultraviolet radiation, and an arid climate; samples for the present study were collected during the local cold-dry season (1 December 2023–29 February 2024), this process yields jerky with a firm texture and distinct flavor, which is popular among local farmers, herdsmen, and tourists [3]. However, the traditional air-drying process results in inconsistent product quality. Standardizing its production necessitates an understanding of flavor formation mechanisms.

Nevertheless, altitude, climate, ambient humidity and native microbial background are tightly confounded in field-sampled jerky from different regions. Consequently, differences in product quality observed in this study reflect regional associations, rather than direct causal effects induced by climate. Air-dried yak meat undergoes a series of biochemical reactions mediated by endogenous enzymes and microorganisms during open-air dehydration; it is often described as spontaneously fermented-like rather than a strictly controlled fermented product [4]. Plateau open-air natural air-drying creates low-temperature, fully aerobic, and progressively dehydrated conditions, which impose strong environmental selection for desiccation-tolerant and psychrotolerant microorganisms. Previous investigations on traditional Xizang naturally air-dried yak jerky and dry-aged beef have documented the frequent predominance of spoilage-related psychrotrophic bacterial genera including *Pseudomonas*, *Brochothrix*, and *Psychrobacter* in these meat matrices [5,6]. Lactic acid bacteria require sufficient fermentable carbohydrates, appropriate salt levels, and micro-anaerobic or anaerobic niches together with suitable fermentation temperatures to achieve numerical dominance; these environmental prerequisites are largely absent under the open-air natural air-drying regime applied in this work. Flavor is one of the most critical quality attributes of dried meat products, and volatile organic compounds (VOCs) constitute the core material basis for the sensory aroma characteristics of jerky. Variations in VOC profiles directly determine the aroma differentiation among different dried meat products. Microorganisms such as lactic acid bacteria, staphyloccci, yeasts, and molds play pivotal roles in flavor development of meat products [7]. For instance, *Lactobacillus* and *Weissella* promote the synthesis of VOC (e.g., alcohols, esters, and acids) in sour meat, thereby improving its flavor quality [8]; similarly, *Micrococcus*, *Lactobacillus*, and *Saccharomyces* cerevisiae drive the production of characteristic flavor substances (e.g., secondary alcohols, ethyl esters, and dimethyl trisulfide) in Parma ham [9]. Although microorganisms are key contributors to flavor formation in fermented-like meat products, their community composition is not constant under practical production conditions. Variations in microbial abundance and taxa significantly affect the microbial succession process and final flavor. Such variations are primarily associated with raw meat quality [10], air-drying environmental conditions (e.g., temperature, humidity, altitude) [11], and maturation time [12]. For five U.S.-based and 17 French dry-aged beef producers, the main factors regulating the diversity and composition of beef jerky microbial communities were producer, temperature, and relative humidity [6,13]. Ribeiro et al. [14] found that under controlled temperature and airflow, different relative humidities (50%, 70%, 85%) did not significantly affect microbial counts during dry aging, though the dominant microbiota differed between dry- and wet-aged beef, with Pseudomonadales prevailing in dry-aged samples.

Recent research on beef jerky production has mostly focused on process optimization and quality characterization. For example, Zhao et al. [15] systematically summarized the processing modes, quality properties and technical development of Chinese beef jerky; Li [16] further reviewed key technical bottlenecks and quality-control strategies for traditional dried beef products. Zhang et al. [17] reported that humidification during hot-air impact drying improves beef jerky quality by mitigating surface crusting, enhancing color, reducing lipid oxidation, and intensifying roasted-dried flavor. However, studies on the associations among climatic conditions, microorganisms, and characteristic flavor (especially VOC-driven aroma differences) during air-dried yak meat preparation remain insufficient. Therefore, this study aimed to characterise microbial community structure and flavor-profile differences in air-dried yak jerky from Nagqu and Nyingchi, and to explore their potential associations with regional environmental factors. Headspace solid-phase microextraction coupled with gas chromatography-mass spectrometry (HS-SPME-GC-MS) was used to determine VOCs in yak jerky from these two Tibetan regions. Additionally, dominant microbial taxa were correlated with flavor substances to reveal potential links driving flavor differences in yak jerky. These findings will provide basic reference information for future quality improvement and stabilization of industrially produced yak jerky, while further controlled experiments are required to verify concrete mechanisms.

## 2. Materials and Methods

### 2.1. Materials and Reagents

Yak tenderloin (psoas major muscle) and round (quadriceps femoris muscle, hind-quarter muscle) meat were obtained from six female yaks (aged 4–5 years) slaughtered at a local abattoir in Jiali County, Nagqu, Tibet Autonomous Region China, in December 2023. The post-mortem time before processing was 24 h. The meat samples were trimmed into strips with dimensions of 12 × 4 × 2 cm (length × width × thickness). Natural air-drying was conducted in Nagqu and Nyingchi under indoor, well-ventilated conditions with natural airflow and no direct sunlight exposure. Meat strips were hung approximately 2 m above the ground with a spacing of 10 cm between strips. The drying period spanned from 1 December 2023 to 29 February 2024, comprising three independent drying batches (one per month) to prepare six sample groups: Nagqu air-dried tenderloin (NFL), Nagqu air-dried round (NFH), Nyingchi air-dried tenderloin (LFL), Nyingchi air-dried round (LFH), as well as Nagqu fresh tenderloin (NYXL) and Nagqu fresh round (NYXH) (served as controls). After drying, all samples were sealed in sterile bags, placed in insulated boxes with ice packs, and transported to the laboratory within 24 h for analysis. The three replicates for each group represent independent biological samples from these three monthly batches, not repeated measurements from the same batch.

DNA extraction kit (Nobleryder, Beijing, China), Universal DNA Purification and Recovery Kit (Tiangen Biotech Co., Ltd., Beijing, China), Phusion High-Fidelity PCR Master Mix with GC Buffer (New England Biolabs, Ipswich, MA, USA), Phusion High-Fidelity DNA Polymerase (New England Biolabs, Ipswich, MA, USA), and 2% agarose (Biowest, Nuaillé, Spain) were used in this study.

### 2.2. Methodology

**Figure 1:**
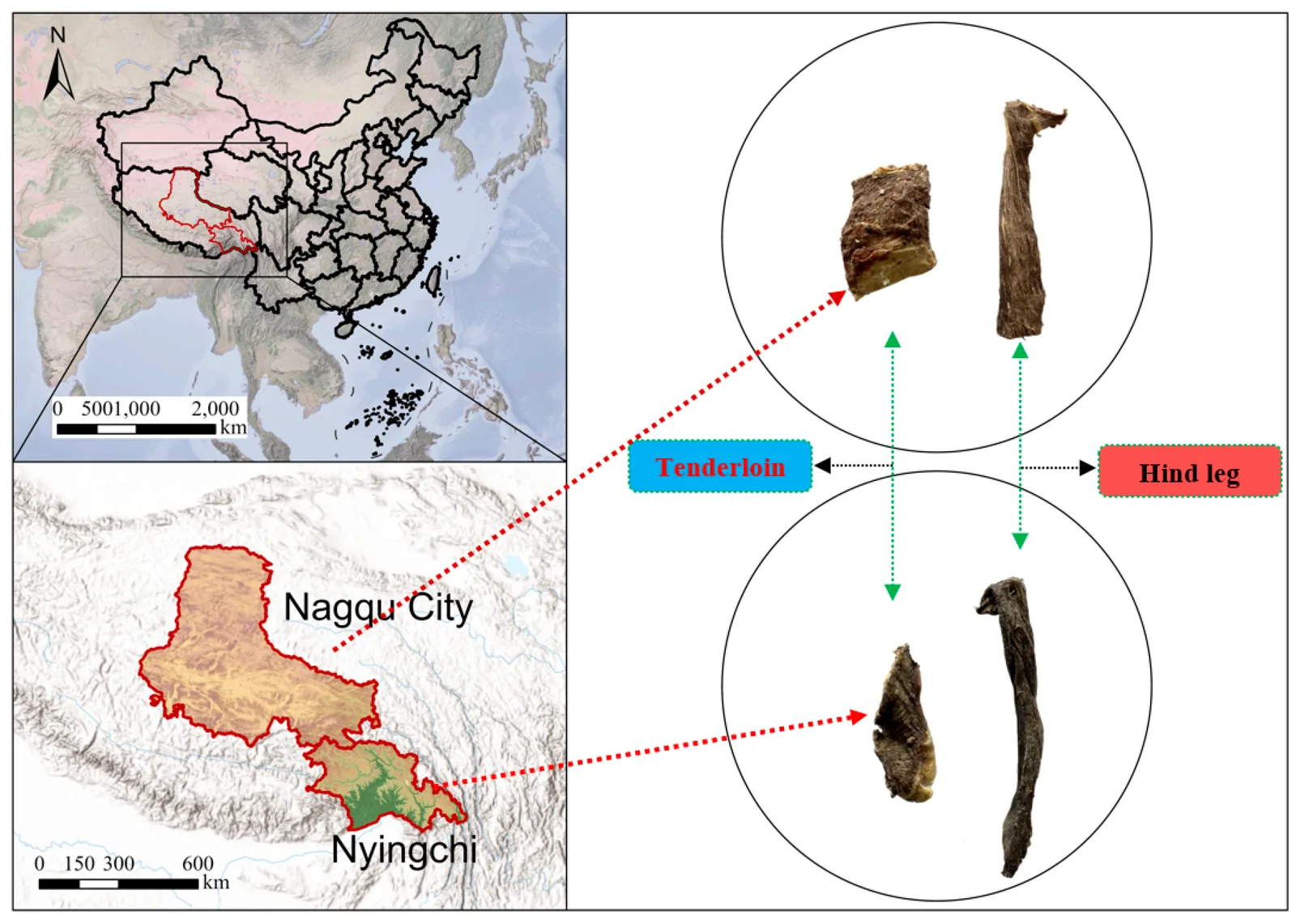
Schematic diagram of yak jerky in different air-drying environments.

The sampling region in Xizang and representative air-dried yak jerky samples are illustrated in Figure 1 (Base Map Approval No.: GS (2024) 0650).

#### 2.2.1. Homogenization Treatment of Yak Jerky

Approximately 5.0 g of yak jerky sample was weighed and cut into small fragments (1–2 mm). The fragmented sample was transferred to a sterile homogenization tube, and 45 mL of sterile phosphate-buffered saline (PBS, pH 7.4) was added to completely submerge the sample. The homogenization tube was then placed in an ice bath, and a tissue homogenizer (Covaris, Inc., Woburn, MA, USA) was used to homogenize the sample at a rotational speed of 5000 rpm. Homogenization was performed in cycles: 60 s of homogenization followed by a 30 s interval, repeated 4 times, until a homogeneous suspension was formed. The ice bath was maintained throughout the homogenization process to ensure the temperature remained at 4 °C and prevent DNA degradation. Finally, the homogenized suspension was transferred to a new sterile centrifuge tube for subsequent DNA extraction.

#### 2.2.2. DNA Extraction and PCR Amplification

For bacterial community analysis, total genomic DNA was extracted from homogenized yak jerky tissue samples using the cetyltrimethylammonium bromide (CTAB) method. DNA concentration and purity were evaluated via electrophoresis on a 1% (*w*/*v*) agarose using Horizontal agarose electrophoresis system (DYCP-32C, Beijing Liuyi Instrument Factory, Beijing, China). Based on the measured concentration, DNA samples were diluted to 1 ng μL^−1^ using sterile ultrapure water. The V3–V4 hypervariable regions of the bacterial 16S rRNA gene were amplified by polymerase chain reaction (PCR) on a PCR system (Bio-Rad Laboratories, Inc., Hercules, CA, USA), using the barcoded specific primers 341F (5′-CCTAYGGGRBGCASCAG-3′) and 806R (5′-GGACTACNNGGGTATCTAAT-3′). All PCR reactions were prepared in a 25 μL total volume (recommended for reproducibility) containing 15 μL of Phusion High-Fidelity PCR Master Mix (New England Biolabs, Ipswich, MA, USA), 2 μM of each forward and reverse primer, and approximately 10 ng of template DNA. The thermal cycling program was as follows: initial denaturation at 98 °C for 1 min; followed by 30 cycles of denaturation at 98 °C for 10 s, annealing at 50 °C for 30 s, and extension at 72 °C for 30 s; and a final extension step at 72 °C for 5 min.

PCR products were mixed with an equal volume of 1× Tris-acetate-EDTA (TAE) buffer and separated by electrophoresis on a 2% (*w*/*v*) agarose. Amplicons were then pooled at equal concentrations and purified using the Universal DNA Purification Kit (Tiangen Biotech Co., Ltd., Beijing, China). Purified amplicons were subjected to library construction and sequenced on a DNA sequencer (Illumina, Inc., San Diego, CA, USA).

For fungal community analysis, the ITS1-ITS2 region of the fungal internal transcribed spacer (ITS) gene was amplified by PCR using the primers ITS5-1737F (5′-GGAAGTAAAAGTCGTAACAAGG-3′) and ITS2-2043R (5′-GCTGCGTTCTTCATCGATGC-3′). Fungal genomic DNA extraction and PCR amplification were performed following the same protocols as described for bacterial DNA sequencing.

#### 2.2.3. pH

Yak jerky samples were homogenized using a tissue homogenizer at 5000 rpm in an ice bath to obtain a uniform paste. To 1.00 ± 0.01 g of the homogenized yak meat, 9.0 mL of distilled water was added. The mixture was vortex-mixed for 30 s, and the pH value was immediately measured using a benchtop pH meter (PHS-3E, Shanghai Leici Instrument Factory, Shanghai, China). The final pH value was reported as the mean ± SD of three parallel replicates.

#### 2.2.4. Moisture Content

Moisture content of yak jerky samples was determined in accordance with the Chinese National Standard GB 5009.3-2016 [18] (Title: Determination of Moisture in Foods), using the direct drying method with an electrothermal constant-temperature forced-air drying oven (TGF-9140A, Shanghai Zhetu Scientific Instrument Co., Ltd., Shanghai, China). specified therein. Each sample was analyzed in triplicate, and the results were expressed as the mean ± SD.

#### 2.2.5. Fat Content

The crude fat content of yak jerky samples was determined via the Soxhlet extraction method using a fat analyzer (S0X606, Hanon Advanced Technology Group Co., Ltd., Jinan, Shandong, China), in strict accordance with the Chinese National Standard GB/T 5009.6-2003 [19] (Title: Determination of Crude Fat in Foods).

#### 2.2.6. Colour

Colorimetric analysis of air-dried yak meat samples was performed using a portable colorimeter (CR-10 Plus, Konica Minolta, Inc., Tokyo, Japan) to measure the CIELAB color parameters, including lightness (L*), redness (a*), and yellowness (b*). Prior to analysis, samples with a uniformly flat surface were selected to ensure consistent contact between the colorimeter’s measuring probe and the sample matrix. For each sample, five distinct, non-overlapping regions were chosen; the colorimeter was scanned across each region in a back-and-forth motion, and measurements were recorded in five parallel replicates. The final L*, a*, and b* values for each sample were reported as the mean ± standard deviation (SD) of the five replicate measurements.

#### 2.2.7. Texture

Yak meat samples were first processed to remove visible fat and connective tissue (fascia). Subsequently, the trimmed samples were cut into cuboids (1.0 cm × 1.0 cm × 1.0 cm) along the direction of muscle fibers to ensure consistency in sample orientation. Samples with obvious defects (e.g., tissue tears, uneven surfaces) were discarded, and texture analysis (TA·Touch, Shanghai Baosheng Industrial Development Co., Ltd., Shanghai, China) was performed immediately after sample preparation to avoid texture deterioration due to environmental factors (e.g., moisture loss).

Five key texture profile analysis (TPA) parameters—hardness, springiness, chewiness, gumminess, and cohesiveness—were measured using a texture analyzer. The measurement conditions were set as follows: Probe type: TA/0.5S cylindrical probe; Pre-test speed: 2 mm·s^−1^; Test speed (in-test speed): 1 mm·s^−1^; Post-test speed: 1 mm·s^−1^; Compression deformation: 50% of the original sample height; Trigger force: 5 g.

Each sample was measured in at least five parallel replicates (at distinct, non-overlapping positions on the sample surface), and the final values for each TPA parameter were reported as the mean ± standard deviation (SD).

#### 2.2.8. Electronic Nose

Volatile odor analysis was performed following the method described by He et al. [20], with minor modifications. Yak jerky samples were homogenized using a tissue homogenizer at 5000 rpm in an ice bath to obtain a uniform paste. Briefly, 2.00 ± 0.01 g of the homogenized yak meat was accurately weighed and transferred into pre-cleaned, individually labeled 10 mL headspace vials, which were immediately sealed with crimp-top caps (with PTFE-lined septa) to prevent volatile loss. The sealed vials were equilibrated in an electrothermal constant-temperature water bath (HH-4, Shanghai Yiheng Scientific Instrument Co., Ltd., Shanghai, China) for 30 min at 45 °C to facilitate the release of volatile compounds into the headspace. Each sample was analyzed in three parallel replicates to ensure data reproducibility. Measurements were conducted using an electronic nose instrument (cNose, Shanghai Baosheng Industrial Development Co., Ltd., Shanghai, China).

The operating parameters for volatile detection were set as follows: Carrier gas: Dry, oil-free compressed air; Carrier gas flow rate: 1.0 L·min^−1^; Detection duration: 120 s; Sample injection time (or “sensor exposure time,” as per instrument protocol): 90 s.

Odor fingerprint data were acquired for each replicate, and the average fingerprint profiles were used for subsequent analysis. The target volatile compounds sensitive to each sensor (i.e., sensor-specific sensitive substances) are listed in the Supplementary Table (Table S1).

#### 2.2.9. Volatile Flavouring Substances

Volatile compound extraction and analysis were performed following the method described by Domínguez et al. [21] with minor modifications. For extraction, 3.50 ± 0.01 g of dried yak meat sample was accurately weighed into a 20 mL glass extraction vial. Subsequently, 70 μL of 2,4,6-trimethylpyridine (internal standard, 0.05 mg·mL^−1^ in methanol) was added, and the vial was immediately sealed with a crimp cap (PTFE-lined septum) to prevent volatile loss. The sealed vial was placed in an 85 °C water bath (magnetic stirring at 500 rpm) for 20 min to equilibrate, after which a solid-phase microextraction (SPME) fiber was inserted into the headspace for volatile adsorption over 30 min. Prior to use, the SPME fiber was conditioned in the GC inlet port at 250 °C for 20 min to eliminate residual contaminants. Solvent blank and fiber blank controls were analyzed in each analytical batch to exclude background contamination.

Gas chromatography-mass spectrometry (GC-MS) analysis using an Agilent 6890N gas chromatograph coupled with a 5973 mass spectrometer (Agilent Technologies, Inc., Santa Clara, CA, USA) was conducted under the following conditions: Inlet temperature: 250 °C; GC-MS interface temperature: 250 °C; Carrier gas: Helium (purity ≥ 99.999%); Carrier gas flow rate: 1.5 mL·min^−1^; Split ratio: 4:1; Oven temperature program: Initial hold at 40 °C for 5 min; ramped at 5 °C·min^−1^ to 250 °C; final hold at 250 °C for 10 min; Ion source temperature: 230 °C; Quadrupole temperature: 150 °C; Ionization mode: Electron ionization (EI, 70 eV); Mass scan range: 35–550 m/z.

Mass spectral data were matched against the NIST 17 mass spectral library, and only compounds with a match quality ≥80% were retained for further analysis. Due to the absence of authentic reference standards and retention index calibration, all volatile compounds were tentatively identified. This quantification was defined as semi-quantitative analysis, because only a single internal standard was applied without compound-specific response factors for calibration. Relative quantification of volatile compounds was performed using the internal standard method (2,4,6-trimethylpyridine) with the following formula:

$$C_{x}\;=\;\frac{A_{x}}{A_{is}}\;\times \;C_{is}\;\times \;V_{is}\;\times \;\frac{100}{M_{x}}$$

where

*C_x_*: Content of the target volatile compound (μg·(100 g)^−1^)

*A_x_*: Chromatographic peak area of the target compound

*A_is_*: Chromatographic peak area of the internal standard

*C_is_*: Concentration of internal standard solution (μg·μL^−1^)

*V_is_*: Volume of the internal standard added (μL)

*M_x_*: Mass of the yak jerky sample (g)

The factor of 100 was used to convert the calculated results into μg per 100 g of sample

### 2.3. Data Processing and Analysis

All experimental data were collated using Microsoft Excel 2016 (Microsoft Corporation, Redmond, WA, USA) and expressed as the mean ± standard deviation (SD). where *n* = 3 represents independent biological replicates. Multiple measurements on one single sample are defined as technical replicates and were averaged prior to statistical analysis. Statistical analyses were performed using SPSS Statistics 26.0 software (IBM Corporation, Armonk, NY, USA). One-way analysis of variance (ANOVA) was used to assess overall differences among groups, followed by Duncan’s multiple range test for post hoc comparisons. Differences were considered statistically significant at *p* < 0.05.

For multivariate statistical analyses, the following methods were applied:

Principal component analysis (PCA): Data were log-transformed and Pareto-scaled prior to analysis. PCA was performed using SIMCA software (version 14.1, Sartorius).

Orthogonal partial least squares discriminant analysis (OPLS-DA): Data were log-transformed and Pareto-scaled. The model was validated using 7-fold cross-validation and a permutation test (*n* = 200). Variables with variable importance in projection (VIP) > 1.0 and *p* < 0.05 from the corresponding t-test were considered significant.

LEfSe analysis: Linear discriminant analysis (LDA) effect size was performed to identify differentially abundant taxa. The threshold for the LDA score was set to 2.0, and the alpha value for the factorial Kruskal–Wallis test was 0.05. Multiple-testing correction was applied using the Benjamini–Hochberg procedure.

Correlation analysis: Pearson correlation was used to evaluate linear relationships between physicochemical parameters and volatile compounds. Notably, microbial relative abundances are compositional data, and Pearson correlation may be influenced by the inherent properties of such data. Therefore, correlations involving microbial data were interpreted with caution, and all causal expressions have been replaced with neutral terms such as “associated with” or “correlated with”. All correlation heatmaps (including intra-group and inter-group) were generated using ChiPlot software (https://www.chiplot.online/; accessed on 1 January 2025) with the following clustering parameters: complete linkage, Euclidean distance, no standardization, column-wise standardization direction.

Amplicon sequencing data were analyzed by Wekemo Tech Group Co., Ltd. (Shenzhen, Guangdong, China) using their online bioinformatics platform (https://www.bioincloud.tech/; accessed on 1 January 2025). The analysis workflow followed Gao et al. [22,23], with minor modifications for microbiological data visualization.

## 3. Results and Discussion

### 3.1. Meteorological Conditions

Meteorological data for Nagqu and Nyingchi were retrieved from the Weather24Details online database (available at: https://www.tianqi24.com/), which aggregates records from local meteorological stations, accessed on 28 March 2024. A summary of these data is presented in Table 1. It should be noted that these monthly records from an online weather platform may not fully capture the actual microclimatic conditions at the specific drying sites; direct on-site monitoring would be ideal but was not performed in this study.

The two regions possess distinct geographical and climatic backgrounds. Nagqu, located in the hinterland of the Qinghai-Xizang Plateau in northern Xizang, serves as the headwater basin for several major rivers (e.g., the Yangtze, Nujiang, Lhasa, and Yarlung Zangbo Rivers). Nyingchi, situated in southeastern Xizang, is known for its relatively mild climate and is often referred to as the “Jiangnan of Xizang.”

As shown in Table 1, Nagqu has a substantially higher average altitude (~4500 m) compared to Nyingchi (~3100 m). Correspondingly, Nagqu exhibits lower ambient temperatures (−8 to −11 °C in winter months), relative humidity (44–57%), and rainfall (0–0.44 mm) than Nyingchi (temperature 1.8–4.2 °C, humidity 53–68%, rainfall 0–1.62 mm). These climatic factors are inherently intertwined with the geographical location and cannot be treated as independent variables. However, given these substantial regional climatic differences, we hypothesized that air-dried yak meat from Nagqu and Nyingchi would show variations in flavor profiles and microbial communities, which are key quality determinants of the final product.

### 3.2. Microflora

#### 3.2.1. Microbiological Diversity

Sequencing libraries were constructed targeting the bacterial 16S rRNA gene V3–V4 hypervariable regions and fungal ITS1–5F regions. Microbial diversity was analyzed across four yak jerky samples (NFL, NFH, LFL, LFH). Diversity indices and sequence statistics are summarized in Table 2. The Shannon diversity index reflects community diversity based on taxon abundance and evenness, with higher values indicating greater bacterial diversity. The Simpson index (calculated as D = Σpi^2^, where pi is the relative abundance of the i-th OTU, using QIIME2 v.2023.5) also measures diversity, with higher values indicating lower diversity [24,25]. Both Chao1 and ACE indices are metrics used to estimate species richness (i.e., the total number of distinct species) in a sample.

**Figure 2:**
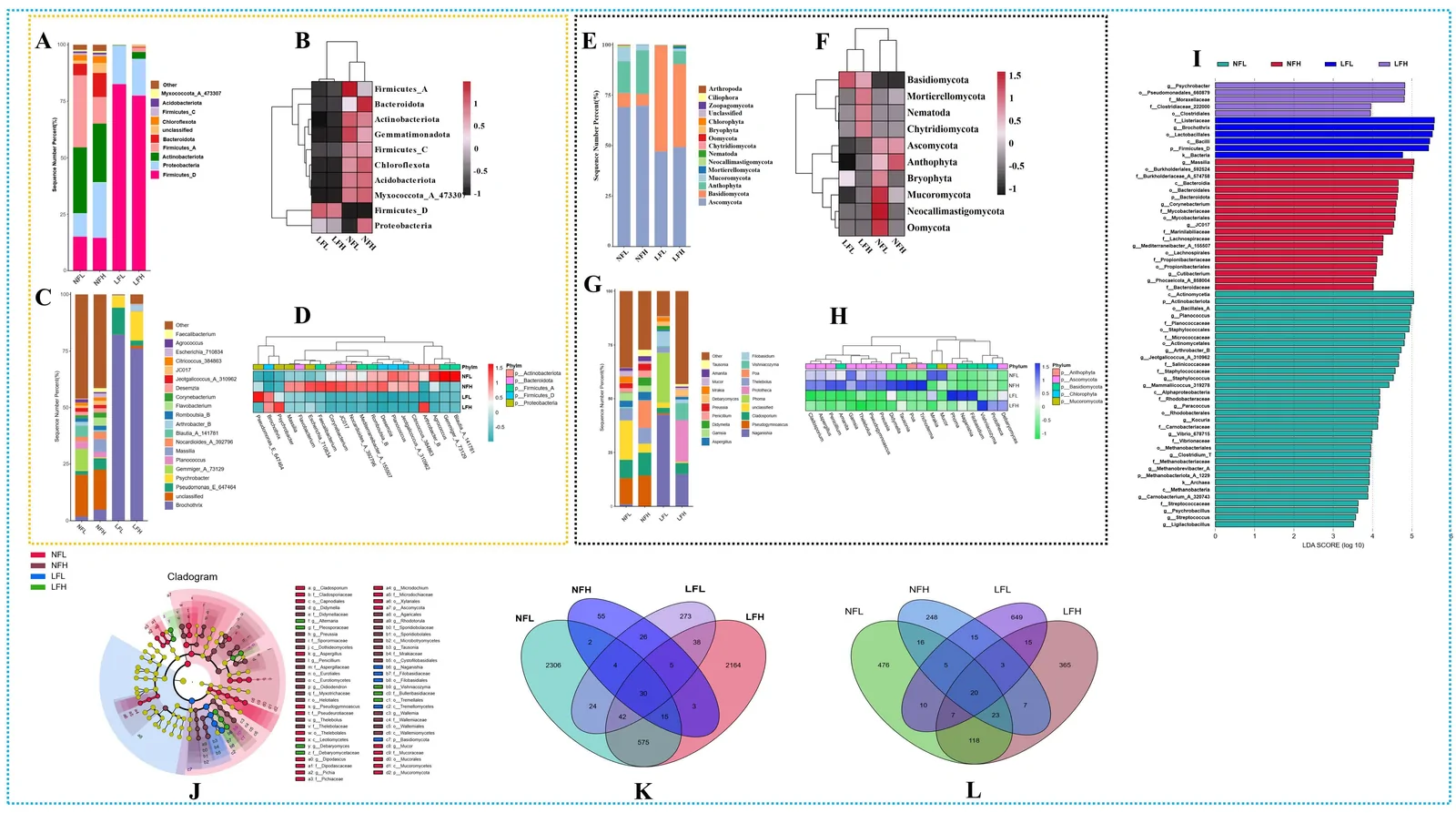
Microorganisms of yak jerky in different regions: Percentage stacking histogram and group clustering heat map of each sample group at the level of bacteria phylum (**A**,**B**), genus (**C**,**D**) and fungal phyla (**E**,**F**) and genus (**G**,**H**); Bacterial (**I**) and fungal (**J**) LEfSe analysis diagrams (the prefixes k, p, c, o, f, and g represent the kingdom, phylum, class, order, family, and genus, respectively); Bacteria (**K**) and fungi (**L**) feature Venn diagrams.

Nagqu samples exhibited higher Simpson, ACE, and Chao1 indices compared to Nyingchi samples, suggesting a trend toward greater community richness in Nagqu; however, formal statistical comparisons were not performed for these α-diversity metrics in the current study. In contrast, Nyingchi samples showed higher Shannon diversity indices, reflecting a more even distribution of bacterial taxa, whereas Simpson index values were also higher in Nagqu, suggesting lower evenness. These results suggest that the α-diversity of microbial communities in yak jerky differed between the two regions. Illumina sequencing generated 403,838 high-quality bacterial reads and 395,491 high-quality fungal reads from the four samples (Table 2). The number of observed operational taxonomic units (OTUs) was 22,692 for bacteria and 853 for fungi. With sequence coverage exceeding 99.99%, these data adequately characterized the microbial communities in the samples. Shared taxa between the two regions included 30 bacterial and 20 fungal taxa, with Nagqu samples exhibiting greater microbial species diversity (Figure 2K,L).

#### 3.2.2. Bacterial Microbiology from Different Regions

The relative abundance of bacterial phyla in Nagqu and Nyingchi yak jerky samples is presented in Figure 2A. A total of 10 bacterial phyla were detected across the four sample groups. Among these, seven phyla had an average relative abundance > 1% in Nagqu samples (NFH, NFL), with five phyla accounting for 89% of all sequenced bacterial reads. For Nyingchi samples (LFL, LFH), five phyla showed an average relative abundance > 1%, and four phyla contributed to 99% of the total bacterial reads. The dominant phyla in Nagqu were Firmicutes (81%, including Firmicutes_A: 1% and Firmicutes_D: 80%), Proteobacteria (15%), and Actinobacteriota (2%). In contrast, Nyingchi samples were dominated by four phyla: Firmicutes (36%, including Firmicutes_A: 14% and Firmicutes_D: 22%), Actinobacteriota (28%), Proteobacteria (17%), and Bacteroidota (8%), with the combined relative abundance of these four phyla reaching 99% of all bacterial reads in Nyingchi samples.

Data standardization via Z-score transformation was performed to visualize dominant bacterial phyla more intuitively (Figure 2B). Firmicutes_D (a subclass of Firmicutes) exhibited higher relative abundance in Nagqu jerky, whereas Bacteroidota and Actinobacteriota were more abundant in Nyingchi samples. Additionally, Proteobacteria showed high relative abundance in the NFH group. Notably, Firmicutes and Proteobacteria are recognized as key bacterial phyla involved in the fermentation and maturation of fermented meat products, contributing to flavor development and microbial safety. Unclassified bacteria were exclusively detected in Nagqu samples (NFL, NFH), with an average relative abundance of 3%. These findings highlight the need to optimize raw meat handling and processing techniques to minimize the risk of harmful microbial contamination from raw materials or the processing environment.

The distribution of bacterial genera across samples is depicted in Figure 2C. Of the 20 identified bacterial genera, the dominant taxa in Nagqu samples were *Gemmiger*_A_73129 (9.5%), *Massilia* (5.6%), *Pseudomonas*_E_647464 (5.1%), and Planococcus (3.1%)-consistent with the observations reported by Lai et al. [26]. Zhong et al. [27] noted that *Gemmiger*_A-like bacteria exhibit probiotic properties, as they produce butyric acid: a metabolite essential for intestinal epithelial cell health, which provides energy to colonocytes and promotes intestinal cell growth and repair. In this study, only high relative abundance of *Gemmiger*_A_73129 was observed. Whether this specific taxon possesses similar probiotic function cannot be confirmed solely based on amplicon sequencing data and remains to be further verified. Ribeiro et al. [14] identified Pseudomonas as a predominant genus in raw beef; however, Ryu et al. [28] emphasized that *Pseudomonas* acts as a major spoilage-associated genus in meat products when its population reaches a threshold density. *Pseudomonas* detected in naturally air-dried yak jerky originates from the surface of raw yak meat and the open-air drying environment. Although most *Pseudomonas* species are typical psychrotrophic spoilage bacteria in chilled fresh meat, certain psychrotolerant *Pseudomonas* members can survive under open-air drying conditions characterised by high altitude and low water availability in Xizang. Furthermore, the differential distribution of bacterial communities among jerky samples was closely associated with physicochemical properties including pH, moisture content and textural attributes. Variations in pH and moisture provide selective pressure for bacterial proliferation, which further shapes community structure; conversely, bacterial metabolic activities may in turn modify tissue pH and moisture-dependent textural characteristics of air-dried yak jerky. In Nyingchi samples, the dominant genera were *Brochothrix* (79%) and *Psychrobacter* (12%). Further analysis revealed high relative abundance of *Gemmiger* in Nagqu NFH samples and *Psychrobacter* in Nyingchi LFH samples (Figure 2D). Collectively, the bacterial community composition of Nyingchi yak jerky was more taxonomically homogeneous compared to that of Nagqu samples. Such genus-level differences are closely related to the quality characteristics of naturally air-dried yak jerky. For instance, the high abundance of *Brochothrix* in Nyingchi samples may affect meat flavor and spoilage risk; *Psychrobacter* is a typical cold-tolerant microorganism adapted to high-altitude open-air drying environments. In Nagqu samples, *Pseudomonas* may bring potential spoilage risk, while *Gemmiger*_A_73129 may contribute metabolite precursors for flavor formation. The distinct dominant-genus distribution partially explains the quality divergence between yak jerky from the two sampling regions.

#### 3.2.3. Fungal Microbiological Composition

For all sequenced fungal reads, after excluding plant-derived contaminant sequences (Anthophyta and Poa), the combined relative abundance of four dominant fungal phyla accounted for 98% of the total fungal community (Figure 2E). These dominant phyla were Ascomycota (69% in Nagqu samples, 48% in Nyingchi samples), Basidiomycota (64% in Nagqu samples, 47% in Nyingchi samples), Anthophyta (19% in Nagqu samples, 3.2% in Nyingchi samples), and Mucoromycota (4.7% in Nagqu samples, 1.3% in Nyingchi samples)-a taxonomic distribution consistent with the findings reported by Liu et al. [29]. At the phylum level, Anthophyta exhibited high relative abundance in NFH samples, Mucoromycota was enriched in NFL samples, and Basidiomycota was more abundant in LFL samples (Figure 2F). Notable differences in fungal genus composition were observed between Nagqu and Nyingchi yak jerky (Figure 2G). The dominant fungal genera in Nagqu samples were *Pseudogymnoascus* (11%), *Cladosporium* (10%), *Poa* (8%), *Thelebolus* (6%), and *Gamsia* (3%). In contrast, Nyingchi samples were dominated by *Naganishia* (33%), *Cladosporium* (6%), *Filobasidium* (5%), and *Vishniacozyma* (2%).

Microorganisms-predominantly bacteria and fungi-play pivotal roles in flavor development during the processing of fermented meat products. Key functional groups include lactic acid bacteria, staphylococci, and micrococci (for bacteria), as well as yeasts and molds (for fungi) [30,31]. Although no common beneficial fermentative microorganisms (e.g., lactic acid bacteria, functional yeasts) were detected in the present study, the unique climatic conditions and high altitude of Xizang (Xizang Autonomous Region, China) contribute to the distinct microbial flora of local yak jerky. Notably, the dominant fungal genera identified herein (*Pseudogymnoascus*, *Poa*, *Thelebolus*, *Naganishia*) are well-documented cryophilic fungal taxa adapted to high-altitude and cold environments, including Antarctica, the Arctic, and other high-elevation regions worldwide [32]. Consistent with these previous reports [6], the dominant microbial genera recovered from the naturally air-dried yak jerky analysed in this work were mostly spoilage-associated taxa, and typical lactic acid bacteria were not detected as dominant members. This represents a normal ecological outcome for this non-starter-inoculated, air-dried meat product, rather than a methodological artifact or processing defect. The prevailing low-temperature, fully aerobic, and progressive dehydration conditions of plateau natural air-drying exert strong selective pressure favouring desiccation-tolerant, psychrotolerant, and spoilage-related bacterial genera such as Pseudomonas, *Brochothrix*, and *Psychrobacter*. In addition, lactic acid bacteria require sufficient fermentable carbohydrates, appropriate salt levels, and micro-anaerobic or anaerobic niches together with suitable fermentation temperatures to achieve numerical dominance; these environmental prerequisites are largely absent under the open-air natural air-drying regime applied in this work.

At the genus level, *Gamsia* showed the highest relative abundance in NFL samples; *Pseudogymnoascus*, *Cladosporium*, *Poa*, and *Thelebolus* were more abundant in NFH samples; *Naganishia* and *Filobasidium* were enriched in LFL samples; and *Vishniacozyma* exhibited higher relative abundance in LFH samples (Figure 2H). These results suggest that the enrichment of dominant microbial taxa in yak jerky may be associated with the meat cut. Furthermore, the microbial community of yak jerky differs from that of other commercially available jerky products primarily due to the unique climatic conditions and altitude of its production region.

#### 3.2.4. LEfSe Analysis of Air-Dried Yak Meat

LEfSe analysis was performed on four independent yak jerky samples (NFL, NFH, LFL, LFH) to identify region-specific bacterial and fungal biomarkers. The LDA score threshold was set at 3.0, with α = 0.05 for the Kruskal-Wallis test followed by pairwise Wilcoxon rank-sum tests. LEfSe internally applies Bonferroni correction, and LDA scores serve as the primary effect size measure.

LEfSe (Linear Discriminant Analysis Effect Size) is a statistical method used to discover and interpret high-dimensional biosignatures [25]. The LDA threshold was set at 3 in this study. The top four characteristic bacteria in each sample group were analysed by LEfSe. These bacteria in LFH were *Psychrobacter*, Pseudomonadales_660879, Moraxellaceae, and Clostridiaceae_222000; those in LFL were Listeriaceae, *Brochothrix*, Lactobacillales, and Bacilli; those in NFH were *Massilia*, Burkholderiales_592524, Burkholderiaceae_A_574758, and Bacteroidia; and those in NFL were Actinomycetia, Actinobacteriota, Bacillales_A, and Planococcus (Figure 2I).

The cladogram is a dendrogram that represents significant differences in yak jerky in different regions by color and circle size. The LEfSe analysis cladogram is presented in Figure 2J. Characteristic fungi in LFH were Vishniacozyma, Bulleribasidiaceae, Tremellales, and Alternaria; those in LFL were Tremellomycetes, Filobasidiaceae, Filobasidiales, and Basidiomycota; those in NFH were Dothideomycetes, *Thelebolus*, Thelebolaceae, and Eurotiales; and those in NFL were Ascomycota, Leotiomycetes, Thelebolales, and *Pseudogymnoascus*. These features may serve as biological indicators to differentiate yak jerky from different regions.

#### 3.2.5. Differential Analysis of Physicochemical Properties

The pH value of meat is a key indicator of its tenderness, as well as a marker of freshness. Alía et al. [23] noted that fresh meat with a pH > 6.6 is typically classified as inferior. In the present study, the pH of air-dried yak jerky ranged from 5.69 to 5.9 (Table 3), with all samples exhibiting a higher pH compared to fresh yak meat. Notably, significant regional differences in pH were observed between Nagqu and Nyingchi jerky samples (*p* < 0.05). This variation may be attributed to microbial metabolism: microorganisms in the meat interact with endogenous enzymes to degrade proteins, potentially producing alkaline metabolites (e.g., amines) that offset organic acid accumulation, thereby elevating pH.

Moisture content is a critical factor influencing core quality attributes of meat products, including firmness, tenderness, and surface appearance [33]. Across all yak jerky samples, the moisture content of hind leg-derived jerky was consistently higher than that of tenderloin-derived jerky, with the lowest moisture content (6.96%) observed in the LFL sample (Table 3). As reported by Patarata et al. [34], reduced moisture content inhibits microbial growth and proliferation by limiting water availability (water activity, a_w_), thereby extending the shelf-life of food products-a key advantage for air-dried jerky, which relies on low moisture for preservation.

Fat content also differed significantly between meat cuts (*p* < 0.05): across all samples, hind leg-derived jerky had lower fat content than tenderloin-derived jerky. The lowest fat content (5.94%) was detected in the NFH sample (Table 3). This cut-specific difference is consistent with muscle physiology, as tenderloin (a non-weight-bearing muscle) typically accumulates more intramyocellular fat than hind leg (a weight-bearing muscle with higher collagen content).

Minor variations were observed in CIELAB color parameters (lightness, L*; redness, a*; yellowness, b*) among samples, but all three parameters were significantly higher in Nagqu jerky than in Nyingchi jerky (*p* < 0.05)-indicating that Nyingchi jerky exhibited a darker overall appearance (Table 4). Variations in meat colour may arise from raw-meat intrinsic properties such as myoglobin content and lipid oxidation status, combined with regional drying micro-climate and complex metabolic activities of surface microbial communities.

Textural properties are direct predictors of the sensory quality of air-dried meat products. Nagqu jerky exhibited significantly higher springiness and cohesiveness than Nyingchi jerky (*p* < 0.05), while hardness, chewiness, and adhesiveness were significantly lower in Nagqu samples (*p* < 0.05) (Table 5). For air-dried jerky, desirable textural characteristics are defined by low hardness and chewiness (ease of mastication) and high springiness (structural resilience). These results suggest that Nagqu jerky displayed textural properties (higher springiness and cohesiveness, lower hardness and chewiness) that are often associated with favorable eating quality, although direct sensory evaluation was not performed.

### 3.3. Correlations Between Environmental Factors and Microbial Community Composition

**Figure 3:**
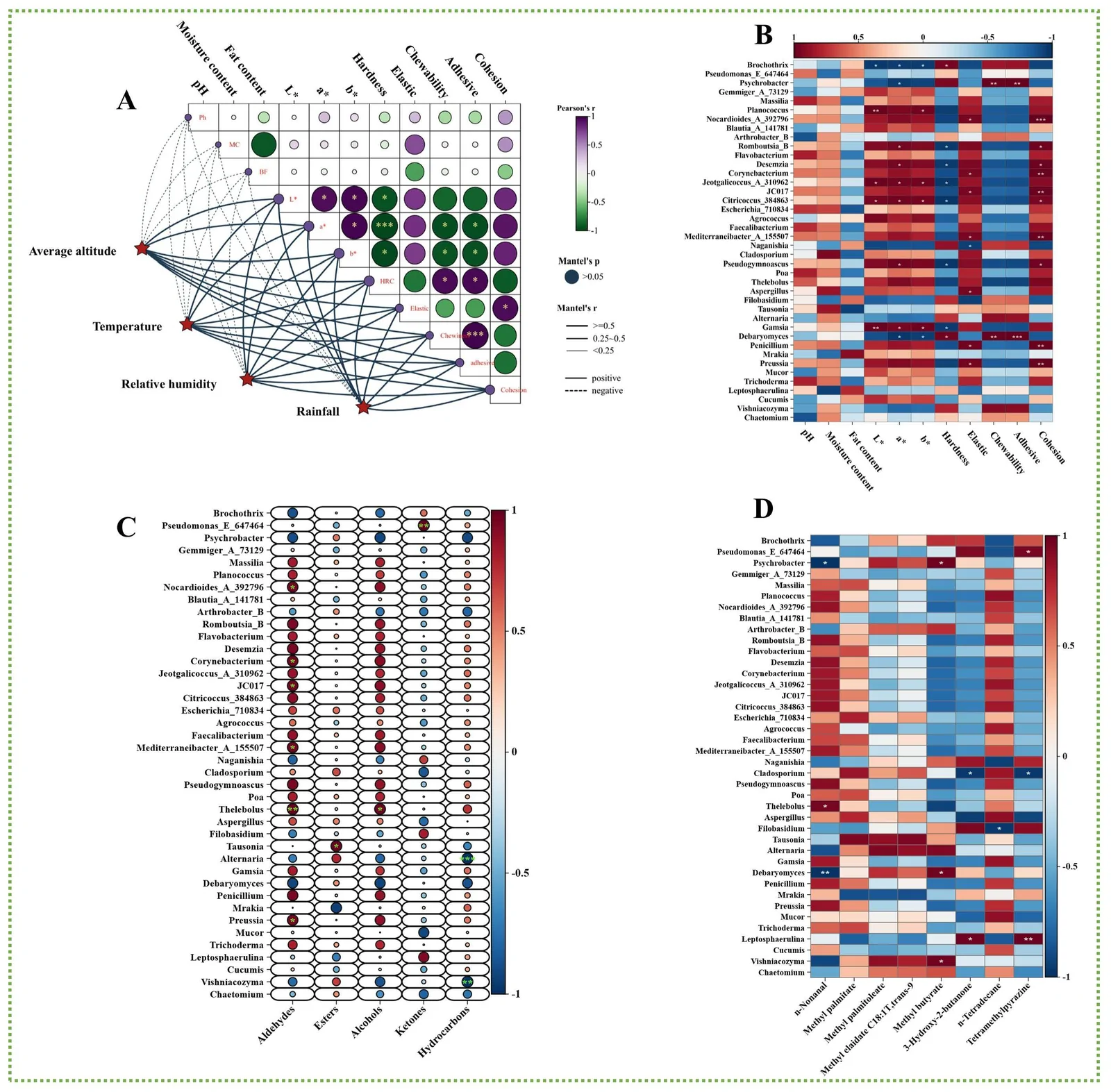
Correlation analysis of yak beef jerky in different regions: Correlation analysis of physical and chemical properties with environmental factors (**A**) and microbial flora (**B**); Correlation analysis of yak beef flavor (**C**) and microorganisms with VIP > 1 and volatile organic compounds (**D**) at the genus level. (Note: * indicates a significant correlation with 0.01 < *p* < 0.05, ** indicates a highly significant correlation with *p* < 0.01, *** indicates an extremely significant correlation with *p* < 0.001. The first 20 microorganisms in the figure are bacterial genera, and the last 20 are fungal genera.

Reduced altitude-accompanied by corresponding increases in temperature, humidity, and rainfall-was associated with changes in yak jerky properties: specifically, positive correlations with *pH*, moisture content, and fat content, and negative correlations with CIELAB color parameters and textural quality (Figure 3A). Analyses of within-sample correlations indicated that jerky samples with high hardness, chewiness, and adhesiveness tended to have a darker appearance. In contrast, cohesion and springiness showed significant positive correlations with color parameters. Microbial community composition further correlated with these physicochemical traits: Jerky samples enriched in the bacterial genera *Brochothrix*, Pseudomonas_E_647464, and, as well as the fungal genera *Naganishia* and Filobasidium, exhibited lower CIELAB color values and higher hardness, chewiness, and adhesiveness (Figure 3B), and was associated with the product’s basic physicochemical properties.

### 3.4. Flavour Variability

#### 3.4.1. Electronic Nose

**Figure 4:**
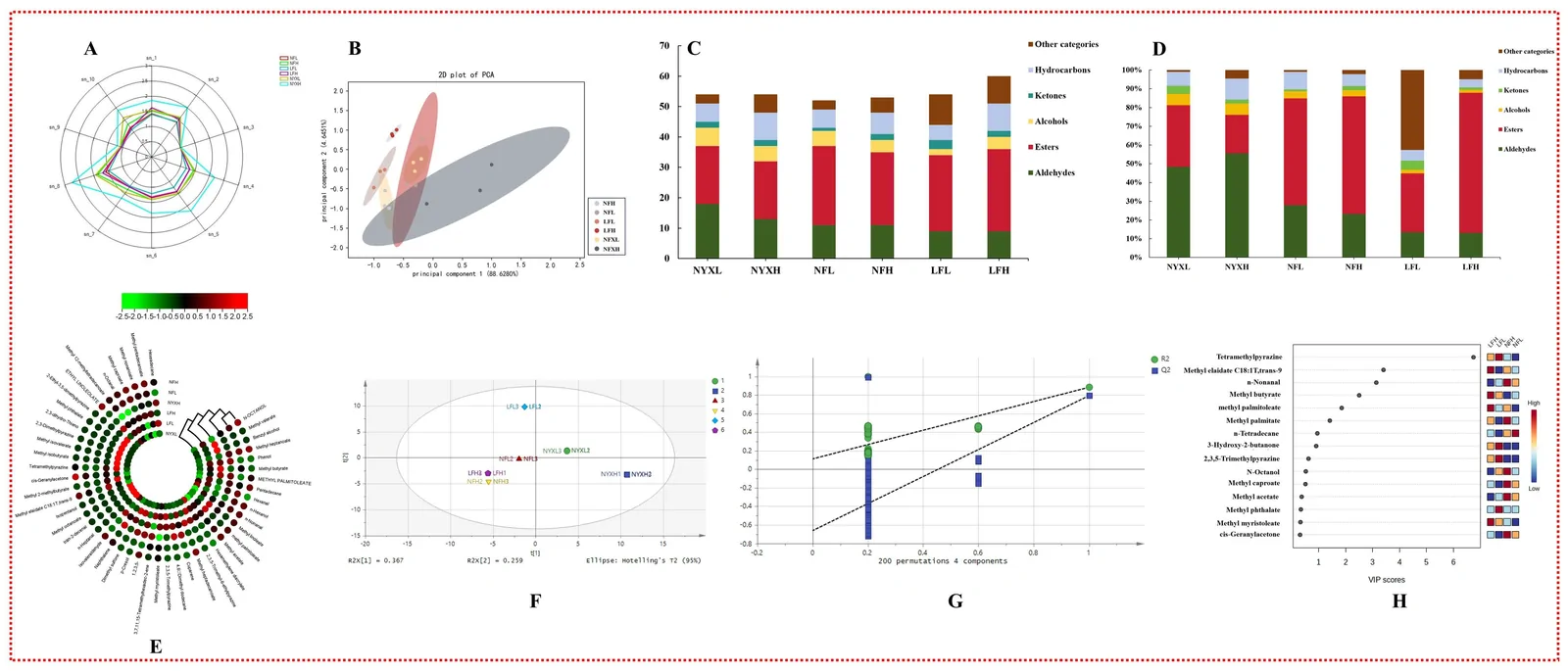
VOC of yak jerky in different regions: analysis of electronic nose of yak jerky ((**A**) radar map; (**B**) PCA); Type (**C**), content (**D**) and cluster heat map (**E**) of VOC in each sample; OPLS-DA analysis of VOC in yak jerky (**F**,**G**) and VIP scores (**H**).

The response results of electronic nose (E-nose) detection are presented in Figure 4A. Although the response profiles of the 10 sensors to volatile compounds in different jerky products were largely similar, among the six meat samples (two fresh yak meat samples and four jerky samples), sensors T2 (Sn_2), T4 (Sn_4), T5 (Sn_5), and T8 (Sn_8) exhibited strong response signals (Table S1). The response values of the sensors for NYXL, NFH, and LFH were higher than those for NFXL, NFL, and LFL, respectively. This suggests that yak meat from the hind leg contains either a higher concentration of volatile compounds or a greater variety and abundance of specific volatile compounds. These results are consistent with those reported by Ueda et al. [35], in which aldehydes, alcohols, sulfur-containing compounds, and aromatic compounds were identified as the primary meat flavor compounds. In contrast, sensors sensitive to ozone, hydrogen-containing gases, and combustible gases exhibited weaker response signals. For sensors T4 (Sn_4), T5 (Sn_5), and T8 (Sn_8), the response values of NFH were significantly higher (*p* < 0.05) than those of NFL, LFH, and LFL, indicating that NFH may contain a greater abundance of volatile compounds.

The contribution of the principal components (PCs) derived from the sensors exceeded 80%, indicating that the analytical results largely encompass the response data of all sensors [36]. In principal component analysis (PCA), the cumulative contribution of PC1 (88.63%) and PC2 (4.65%) reached 93.28%, which exceeded 80% (Figure 4B). This indicates that these two components largely capture all the sample information, which is consistent with the findings reported by Li et al. [37]. Fresh yak meat samples and Nagqu air-dried jerky samples were clustered more densely, suggesting that the flavor of Nagqu yak jerky more closely resembles that of fresh yak meat. However, Nyingchi air-dried jerky samples and fresh Nagqu yak meat samples exhibited greater separation, indicating more pronounced flavor differences between these two sample groups. Although electronic nose-based instrumental analysis can distinguish flavor differences among yak jerky products processed in different environments, it is unable to accurately identify the specific flavor-active compounds responsible for these differences. Therefore, to further characterize the volatile profile of yak jerky, headspace solid-phase microextraction coupled with gas chromatography-mass spectrometry (HS-SPME-GC-MS) was employed to identify and quantify its volatile compounds.

#### 3.4.2. Volatile Flavour Substances

Volatile organic compound (VOC) were analyzed and identified via headspace gas chromatography-mass spectrometry (HS-GC-MS). A total of 94 VOCs were detected, belonging to six chemical classes: 18 aldehydes, 37 esters, 8 alcohols, 3 ketones, 12 hydrocarbons, and 16 compounds categorized as “others” (Table S2). Stacked bar charts were constructed to visualize the distribution of VOC classes (Figure 4C) and their respective contents (Figure 4D). Aldehydes and esters accounted for the largest proportions of VOC across all four jerky types; relative quantification via the internal standard method revealed that NFH had the highest aldehyde content (511.24 ± 0.42 μg/100 g), while LFH exhibited the highest ester content (1617.02 ± 0.96 μg/100 g) (Figure 4C,D). Notably, ester contents were significantly elevated (*p* < 0.05) in all jerky samples compared with fresh yak meat samples (NYXL, NYXH). Relative to fresh meat, the total VOC contents in jerky generally increased, with the highest value observed in LFL (2560.90 ± 2.13 μg/100 g). Esters were identified as the primary contributors to jerky flavor (Table 6).

Esters are primarily synthesized via the hydrolysis of fats and proteins, followed by the esterification of generated alcohols and fatty acids. Due to their low sensory threshold, esters make a significant contribution to the flavor of fermented meat products [38]. Two lactones were detected in this study, among which the formation of γ-butyrolactone is known to play an important role in shaping the aroma of air-dried meat [19]. Compared with fresh meat, only γ-butyrolactone content was significantly increased (*p* < 0.05) in LFL, reaching a peak of 9.18 ± 0.05 μg/100 g. Additionally, contents of flavor-active compounds (e.g., methyl caproate, methyl caprate, methyl laurate, methyl myristate, and methyl palmitate) in jerky were significantly higher (*p* < 0.05) than those in the original fresh meat.

Aldehyde contents are closely associated with lipid oxidation [39]. Hexanal, a well-recognized indicator of lipid oxidation, imparts an undesirable acidic odor at high concentrations but contributes a pleasant grassy note at low concentrations. Substances such as heptanal and octanal—generated during lipid oxidation—also contribute to meat flavor [21]. Hexanal was not detected in NFH and LFH, but its content increased significantly (*p* < 0.05) in NFL and LFL, with the highest level observed in NFL (11.05 ± 0.03 μg/100 g). Compared with fresh Nagqu yak meat (NFXL, NFXH), octanal contents were also significantly elevated (*p* < 0.05) in jerky, peaking in LFL (35.68 ± 0.06 μg/100 g).

Alcohols are mainly produced via the degradation of unsaturated fatty acids catalyzed by enzymes such as lipoxygenases and peroxidases. Many of these alcohols exhibit fruity, vegetal, or floral aromas and can enhance the volatile flavor profile of meat [40]. The primary alcohols detected in jerky samples were hexanol, 1-octen-3-ol, n-octanol, and lauryl alcohol. Among these, 1-octen-3-ol has a very low sensory threshold and can impart a subtle undesirable mushroom-like flavor to meat products. Compared with fresh meat, 1-octen-3-ol contents exhibited a decreasing trend in all jerky samples, with the highest content among jerky observed in NFH (10.77 ± 0.05 μg/100 g).

Ketones are formed via lipid oxidation; most exhibit fruity or creamy aromas—more pronounced than those of aldehydes—and exert a positive effect on the volatile flavor of yak jerky [41]. The highest ketone content was found in LFL (124.13 μg/100 g), while the lowest was observed in NFL (14.54 μg/100 g). Compared with fresh meat, hydrocarbon contents were elevated in tenderloin jerky (NFL, LFL) but reduced in hind-leg jerky (NFH, LFH). Among the “other” compound classes, pyrazines were the most diverse and abundant heterocyclic compounds, with relatively high levels detected in Nyingchi yak jerky. The heatmap (Figure 4E) showed that the samples were clustered into five distinct groups: NFL and NFH in Class I, NYXH in Class II, LFH in Class III, LFL in Class IV, and NYXL in Class V. These results indicate that yak jerky samples from the same production region exhibit similar flavor-active compound profiles, whereas differences exist between samples derived from different muscle cuts.

#### 3.4.3. Air-Dried Yak Meat OPLS-DA

VOCs in dried yak meat were further analyzed using orthogonal partial least squares discriminant analysis (OPLS-DA) (Figure 4F). For the constructed OPLS-DA model, the goodness-of-fit coefficient for the independent variable was R^2^X = 0.93, while that for the dependent variable was R^2^Y = 0.80; the predictive ability index was Q^2^ = 0.61. Since R^2^ and Q^2^ values were close to 1, the model exhibited high goodness-of-fit and predictive accuracy. As shown in Figure 4F, samples from different jerky groups were well-separated in distinct quadrants, confirming the model’s effectiveness in distinguishing sample categories.

To validate the model against overfitting, a permutation test was performed by randomly shuffling the categorical Y matrix 200 times. The permutation results yielded a Q^2^ range of (0.0, −0.635), with the intercept of the regression line for Q^2^ values below 0 (Figure 4G). These findings indicate that the original OPLS-DA model was not overfitted and could be reliably used to identify the characteristic flavor-active compounds of yak jerky. Based on the validated OPLS-DA model, eight VOCs with variable importance in projection (VIP) scores > 1 were screened as potential characteristic flavor-active compounds (Figure 4H): 3-hydroxy-2-butanone, n-tetradecane, methyl palmitate, methyl palmitoleate, methyl butyrate, n-nonanal, methyl elaidate (C18:1T, trans-9), and tetramethylpyrazine. Among these, methyl butyrate, n-nonanal, methyl elaidate (C18:1T, trans-9), and tetramethylpyrazine had VIP scores > 2, indicating they were the most influential in distinguishing sample groups. Notably, tetramethylpyrazine exhibited the highest abundance in LFL.

#### 3.4.4. Correlations Between Microorganisms and Volatile Flavour Substances

Microbial fermentation of carbohydrates in fermented meat products generates diverse flavor-active compounds, including acids, aldehydes, and alcohols. The relationship between microbial communities and flavor profiles was investigated via a correlation heatmap (Figure 3C). Specifically, the bacterial genera *Nocardioides*_A_392796, *Corynebacterium*, JC017, and *Mediterraneibacter*_A_155507, as well as the fungal genera *Thelebolus* and *Preussia,* were associated with yak jerky flavor primarily by increasing aldehyde contents. The fungal genus *Tausonia* exhibited a positive correlation with esters, while *Thelebolus* showed a positive association with alcohol formation, and *Pseudomonas*_E_647464 was correlated with ketone production. Additionally, the fungal genera *Alternaria* and *Vishniacozyma* showed a significant negative correlation with hydrocarbon formation (*p* < 0.05). Further, the fungus *Thelebolus* facilitated the production of n-nonanal; the bacterium *Psychrobacter* and fungi *Debaryomyces* and *Vishniacozyma* promoted methyl butyrate formation; the fungus *Leptosphaerulina* significantly increased 3-hydroxy-2-butanone production; and both the bacterium *Pseudomonas*_E_647464 and fungus *Leptosphaerulina* displayed a significant positive correlation with tetramethylpyrazine levels (*p* < 0.05) (Figure 3D). Collectively, these results reveal associations between dominant microbial genera (e.g., *Thielavia*, *Pseudomonas_E_647464*, *Brochothrix*, *Naqeshwaromyces*) and key volatile compounds, including aldehydes, esters, ketones, and heterocyclic compounds.

The association between flavor substances and microorganisms is mainly observed through two core pathways: amino acid catabolism and lipid oxidation. *Corynebacterium*, a genus significantly associated with VOCs, converts methionine into methanethiol through sulfur metabolism and further generates sulfur-containing compounds such as dimethyl sulfide, enhancing the meaty aroma characteristics [42]. The lipase of *Debaryomyces* can decompose triglycerides into unsaturated fatty acids such as oleic acid and linoleic acid. These fatty acids can be converted into short-chain fatty acids (e.g., caproic acid) through β-oxidation, or into hexanal, octanal, etc., through autoxidation Domínguez et al. [39]. The relatively high temperature in the Nyingchi area may be associated with increased lipase activity, resulting in the total amount of esters in LFH (1617.02 μg/100 g) being significantly higher than that in the Nagqu samples. While the low-temperature environment in Nagqu may suppress lipid oxidation, the higher abundance of aldehydes in NFH (511.24 μg/100 g) was mainly derived from amino acid catabolism pathways rather than lipid oxidation. In addition, aldehydes produced by amino acid catabolism and ketones generated by lipid oxidation (such as 3-hydroxy-2-butanone) can interact with each other to form a complex flavor network [40]. The association between microorganisms and flavor does not exist in isolation, but rather forms a dynamic interaction with regional terroir conditions (e.g., temperature, humidity, altitude). Research has confirmed that the local microbial community (e.g., lactic acid bacteria, coagulase-negative staphylococci, *Debaryomyces*) is the core link connecting the regional environment and product quality, and its metabolic activity is selectively regulated by terroir factors such as temperature and humidity [39,43]. Palavecino Prpich et al. [44] studied the fermentation process of Harbin dry sausage and found that the abundances of local fungi such as *Aspergillus pseudoglaucus* and *Debaryomyces hansenii* were significantly positively correlated with characteristic flavor substances such as phenethyl alcohol and ethyl octanoate, and their metabolic activity was regulated by environmental humidity. This corresponds to the result in this study that the relatively high humidity in the Nyingchi area may promote the proliferation of fungi such as *Naganishia*, thereby affecting the synthesis of esters (Figure S1). It should be noted that the biomarker taxa identified by LEfSe provide an initial characterization of region-specific microbial differences and would benefit from confirmation with larger sample sets in future studies.

## 4. Conclusions

This study investigated the physicochemical properties, volatile organic compounds (VOCs), and microbial communities of naturally air-dried yak jerky from Nagqu and Nyingchi, Xizang. High-throughput sequencing revealed regional differences in microbial composition: Nagqu jerky was dominated by bacterial genera (*Gemmiger*_A_73129, *Massilia*) and fungal genera (*Pseudogymnoascus*, *Thelebolus*), while Nyingchi jerky featured *Brochothrix*, *Psychrobacter*, and *Naganishia*. HS-SPME-GC-MS identified 94 VOCs, with esters as the primary flavor contributors; Nagqu had higher aldehydes, and Nyingchi higher esters. Correlation and OPLS-DA analyses suggested an association between dominant microbes (e.g., *Pseudomonas*_E_647464, *Thelebolus*) and key VOCs, which may be linked to amino acid catabolism and lipid-oxidation-related pathways. Terroir (temperature, humidity) is associated with microbe-flavor interactions. These observations provide baseline reference information for understanding naturally air-dried yak jerky. Notably, the observed correlations between VOCs and the detected microbial groups can only partially represent the actual microbial activity involved in aroma formation. As this work analysed only four air-dried samples, it should be regarded as a preliminary characterisation; the number of samples needs to be enlarged in further studies.

From a microbiological safety perspective, the detection of spoilage-associated and putative opportunistic pathogenic taxa in naturally air-dried yak jerky does not necessarily imply that the product is unsafe. The traditional air-drying process preserves the meat through several combined hurdles: the marked reduction in moisture content (6.96–9.21% in the present study) and the associated decrease in water availability, the low prevailing temperature during the cold-dry season, and intense ultraviolet radiation at high altitude, all of which restrict the proliferation of undesirable bacteria. The natural decrease in pH during drying, together with antimicrobial metabolites produced by certain indigenous microorganisms, may provide additional protective effects. It should be acknowledged, however, that spoilage bacteria such as *Pseudomonas* and *Brochothrix* can still cause quality deterioration if the product is exposed to temperature abuse or improper storage. Because the present work relied on amplicon sequencing, which does not distinguish viable cells from extracellular DNA and does not resolve strain-level pathogenic potential, these taxa should be regarded as putative rather than confirmed hazards, and their viability and safety relevance remain to be verified by culture-dependent and absolute-quantification analyses.

## Figures and Tables

**Table 1:** Meteorological data: monthly average temperature, relative humidity, and rainfall for Nagqu and Nyingchi, Xizang, 2023–2024.

Xizang	Nagqu Air-Dried			Nyingchi Air-Dried		
Year	2023	2024	2024	2023	2024	2024
Month	December	January	February	December	January	February
Temperature (℃)	−8.0	−11.0	−8.0	4.2	1.8	3.3
Relative humidity (%)	56	57	44	63	68	53
Rainfall (mm)	0.05	0.44	0.00	1.14	1.62	0.77

Nagqu sampling site: average altitude 4500 m; Nyingchi sampling site: average altitude 3100 m. Meteorological data were obtained from local meteorological stations.

**Table 2:** Representative sequences and microbial diversity of yak jerky.

Microbiological	Sample	Sequence Number	Diversity Index				
			OTU	Shannon Index	Simpson Index	ACE Index	Chao1 Index
Bacterium	NFL	123164	1282.33	1.79	0.99	1283.11	1282.74
	NFH	110330	1138.67	1.26	0.98	1139.28	1139.22
	LFL	89721	76.33	8.15	0.32	76.43	76.33
	LFH	80623	194.67	8.56	0.41	194.93	194.69
Fungus	NFL	89038	323.00	5.94	0.96	325.84	326.65
	NFH	93360	211.33	5.43	0.96	214.91	216.73
	LFL	99043	131.67	3.79	0.85	132.65	133.44
	LFH	114050	186.67	4.64	0.89	187.75	187.67

The sequencing coverage of all samples was 100%.

**Table 3:** Differences in physicochemical properties of dried yak meat.

Physicochemical Property	NYXL	NYXH	NFL	NFH	LFL	LFH
pH	5.48 ± 0.02 ^d^	5.51 ± 0.02 ^d^	5.69 ± 0.05 ^c^	5.9 ± 0.02 ^a^	5.82 ± 0.07 ^b^	5.7 ± 0.04 ^c^
Moisture content (%)	70.37 ± 1.13 ^b^	73.48 ± 1.25 ^a^	8.18 ± 0.28 ^cd^	9.21 ± 0.11 ^c^	6.96 ± 0.15 ^d^	9.11 ± 0.19 ^c^
Fat content (%)	2.98 ± 0.20 ^e^	1.15 ± 0.41 ^f^	12.82 ± 0.70 ^b^	5.94 ± 0.07 ^d^	14.46 ± 0.27 ^a^	7.83 ± 0.27 ^c^

Different lower-case letters within a row indicate significant differences between groups (*p* < 0.05).

**Table 4:** Differences in color parameters of dried yak meat.

Color Parameter	NYXL	NYXH	NFL	NFH	LFL	LFH
L*	38.02 ± 2.91 ^ab^	37.04 ± 3.24 ^b^	39.66 ± 2.88 ^a^	37.43 ± 1.50 ^ab^	32.67 ± 3.04 ^c^	32.25 ± 1.10 ^c^
a*	7.07 ± 1.90 ^a^	5.97 ± 1.04 ^b^	5.49 ± 0.34 ^b^	5.47 ± 0.41 ^b^	3.35 ± 0.85 ^c^	2.47 ± 0.42 ^c^
b*	9.98 ± 1.14 ^c^	9.73 ± 0.80 ^c^	15.12 ± 1.05 ^a^	14.24 ± 0.82 ^a^	11.33 ± 1.91 ^b^	10.17 ± 0.32 ^c^

Different lower-case letters within a row indicate significant differences between groups (*p* < 0.05).

**Table 5:** Differences in texture profile analysis (TPA) parameters of dried yak meat.

Texture Parameter	NYXL	NYXH	NFL	NFH	LFL	LFH
Hardness (N)	476.35 ± 62.81 ^c^	131.54 ± 14.31 ^c^	37.23 ± 1.21 ^c^	36.00 ± 0.36 ^c^	3927.85 ± 1349.08 ^b^	5379.69 ± 810.20 ^a^
Springiness (mm)	0.55 ± 0.04 ^c^	0.49 ± 0.07 ^c^	0.76 ± 0.10 ^ab^	0.88 ± 0.10 ^a^	0.53 ± 0.21 ^c^	0.64 ± 0.32 ^ab^
Chewiness (mJ)	150.34 ± 7.55 ^b^	37.07 ± 12.44 ^b^	35.13 ± 8.25 ^b^	61.63 ± 34.52 ^b^	479.02 ± 436.57 ^b^	1000.93 ± 473.25 ^a^
Gumminess(N)	284.07 ± 11.55 ^b^	73.88 ± 16.13 ^b^	45.54 ± 5.14 ^b^	67.74 ± 33.64 ^b^	795.07 ± 421.51 ^b^	1597.23 ± 510.37 ^a^
Cohesiveness	0.53 ± 0.04 ^bc^	0.56 ± 0.09 ^bc^	1.22 ± 0.10 ^b^	1.88 ± 0.93 ^a^	0.19 ± 0.06 ^c^	0.22 ± 0.09 ^c^

Different lower-case letters within a row indicate significant differences between groups (*p* < 0.05).

**Table 6:** The important VOC contents of yak jerky.

Compound Name	CAS	Retention Time/min	Relative Content/μg/100 g					
			NYXL	NYXH	NFL	NFH	LFL	LFH
Acetaldeyde	000075-07-0	4.10	3.71 ± 0.02 ^a^	ND	ND	ND	ND	2.61 ± 0.03 ^b^
Hexanal	000066-25-1	10.96	6.39 ± 0.04 ^c^	5.54 ± 0.04 ^d^	11.05 ± 0.03 ^a^	ND	8.13 ± 0.03 ^b^	ND
n-Octanal	000124-13-0	17.25	14.30 ± 0.03 ^e^	ND	34.23 ± 0.19 ^b^	29.07 ± 0.06 ^c^	35.68 ± 0.06 ^a^	16.70 ± 0.11 ^d^
n-Nonanal	000124-19-6	20.28	20.19 ± 0.03 ^f^	173.62 ± 0.0 ^d^	250.31 ± 0.0 ^b^	271.36 ± 0.0 ^a^	195.68 ± 0.2 ^c^	117.50 ± 0.1 ^e^
Methyl caproate	000106-70-7	14.02	5.13 ± 0.04 ^f^	12.28 ± 0.04 ^e^	50.28 ± 0.03 ^b^	58.59 ± 0.04 ^a^	28.31 ± 0.10 ^d^	34.61 ± 0.09 ^c^
Methyl decanoate	000110-42-9	25.47	55.86 ± 0.10 ^d^	20.14 ± 0.10 ^e^	56.51 ± 0.31 ^c^	61.17 ± 0.22 ^b^	65.83 ± 0.07 ^a^	56.38 ± 0.05 ^c^
Gamma-Butyrolactone	000096-48-0	26.76	7.93 ± 0.04 ^b^	6.78 ± 0.07 ^c^	4.58 ± 0.04 ^f^	6.33 ± 0.11 ^d^	9.18 ± 0.05 ^a^	5.49 ± 0.02 ^e^
Methyl dodecanoate	000111-82-0	30.31	7.55 ± 0.06 ^e^	ND	24.33 ± 0.05 ^d^	35.34 ± 0.07 ^a^	29.55 ± 0.42 ^b^	27.18 ± 0.07 ^c^
Methyl myristate	000124-10-7	34.62	16.28 ± 0.04 ^e^	9.75 ± 0.10 ^f^	58.70 ± 0.03 ^c^	90.90 ± 0.07 ^a^	57.77 ± 0.08 ^d^	84.96 ± 0.08 ^b^
Methyl myristoleate	056219-06-8	35.37	10.33 ± 0.05 ^c^	ND	9.28 ± 0.03 ^d^	10.41 ± 0.03 ^c^	18.12 ± 0.09 ^b^	23.07 ± 0.06 ^a^
Methyl palmitate	000112-39-0	38.56	30.39 ± 0.16 ^f^	52.69 ± 0.04 ^e^	211.66 ± 0.2 ^c^	427.64 ± 0.0 ^a^	127.08 ± 0.0 ^d^	383.08 ± 0.0 ^b^
methyl palmitoleate	001120-25-8	39.06	32.17 ± 0.05 ^f^	47.81 ± 0.11 ^d^	46.58 ± 0.03 ^e^	88.0 ± 0.05 ^b^	56.38 ± 0.29 ^c^	150.56 ± 0.0 ^a^
Methyl stearate	000112-61-8	42.19	3.04 ± 0.08 ^f^	10.70 ± 0.03 ^e^	32.53 ± 0.06 ^c^	72.60 ± 0.03 ^a^	18.02 ± 0.10 ^d^	60.86 ± 0.09 ^b^
Methyl elaidate C18:1T,trans-9	001937-62-8	42.55	12.02 ± 0.04 ^f^	35.80 ± 0.13 ^e^	84.84 ± 0.18 ^c^	189.25 ± 0.1 ^b^	58.18 ± 0.04 ^d^	300.09 ± 0.7 ^a^
Methyl acetate	000079-20-9	5.00	ND	17.80 ± 0.19 ^e^	40.42 ± 0.09 ^b^	65.09 ± 0.02 ^a^	33.16 ± 0.04 ^c^	32.35 ± 0.17 ^d^
Methyl butyrate	000623-42-7	8.06	ND	ND	22.83 ± 0.08 ^c^	ND	47.69 ± 0.27 ^b^	134.58 ± 0.0 ^a^
Methyl phthalate	000131-11-3	40.16	ND	ND	ND	ND	64.21 ± 0.31 ^a^	ND
N-Octanol	000111-87-5	24.55	17.48 ± 0.03 ^e^	28.88 ± 0.11 ^d^	37.65 ± 0.06 ^c^	51.07 ± 0.06 ^a^	38.51 ± 0.1 ^b^	15.44 ± 0.07 ^f^
3-Hydroxy-2-butanone	000513-86-0	17.71	36.6 ± 0.30 ^b^	29.30 ± 0.03 ^c^	ND	ND	104.85 ± 0.0 ^a^	14.76 ± 0.09 ^d^
cis-Geranylacetone	003879-26-3	31.50	1.83 ± 0.09 ^e^	6.09 ± 0.04 ^d^	14.54 ± 0.24 ^b^	45.28 ± 0.09 ^a^	6.19 ± 0.04 ^d^	11.91 ± 0.04 ^c^
n-Tetradecane	000629-59-4	20.70	ND	ND	68.65 ± 0.06 ^a^	53.62 ± 0.08 ^b^	ND	36.11 ± 0.07 ^c^
Tetramethylpyrazine	001124-11-4	22.85	ND	ND	ND	6.09 ± 0.05 ^c^	937.85 ± 0.0 ^a^	30.22 ± 0.53 ^b^
2,3,5-Trimethylpyrazine	014667-55-1	21.04	ND	ND	ND	ND	51.46 ± 0.09 ^a^	19.44 ± 0.06 ^b^

Note: “ND” indicates that the corresponding value was not found or could not be calculated. Values with different superscript lowercase letters within the same column indicate significant differences at *p* < 0.05.

## Data Availability

The raw data presented in this study are available upon reasonable request from the corresponding author.

## Supplementary Materials

The following supporting information can be downloaded at: https://www.mdpi.com/article/10.3390/microorganisms14092058/s1, Supplementary Material S1. Flavor Substance Peak Diagram (Figure S1). Supplementary Material S2. Types of sensitive substances for sensors (Table S1). Supplementary Material S3. VOCs contents of yak jerky (Table S2).
