# Supplementary material for: High-Throughput Amplicon Sequencing Microbial Communities and Volatile Flavour Compounds of Naturally Air-Dried Yak Jerky from Two Regions of Xizang
Source: Microorganisms. 2026 Sep 15;14(9):2058. doi: 10.3390/microorganisms14092058 (PMC13608975; doi:10.3390/microorganisms14092058)
Supplement: Supplementary file 1 [file microorganisms-14-02058-s001.zip › microorganisms-4507821-supplementary.pdf]

**Supplementary Material S1.** Flavor Substance Peak Diagram.（Figure S1）

**Supplementary Material S2.** Types of sensitive substances for sensors (Table S1).

**Supplementary Material S3.** VOCs contents of yak jerky (Table S2).

**Supplementary Material S1.**

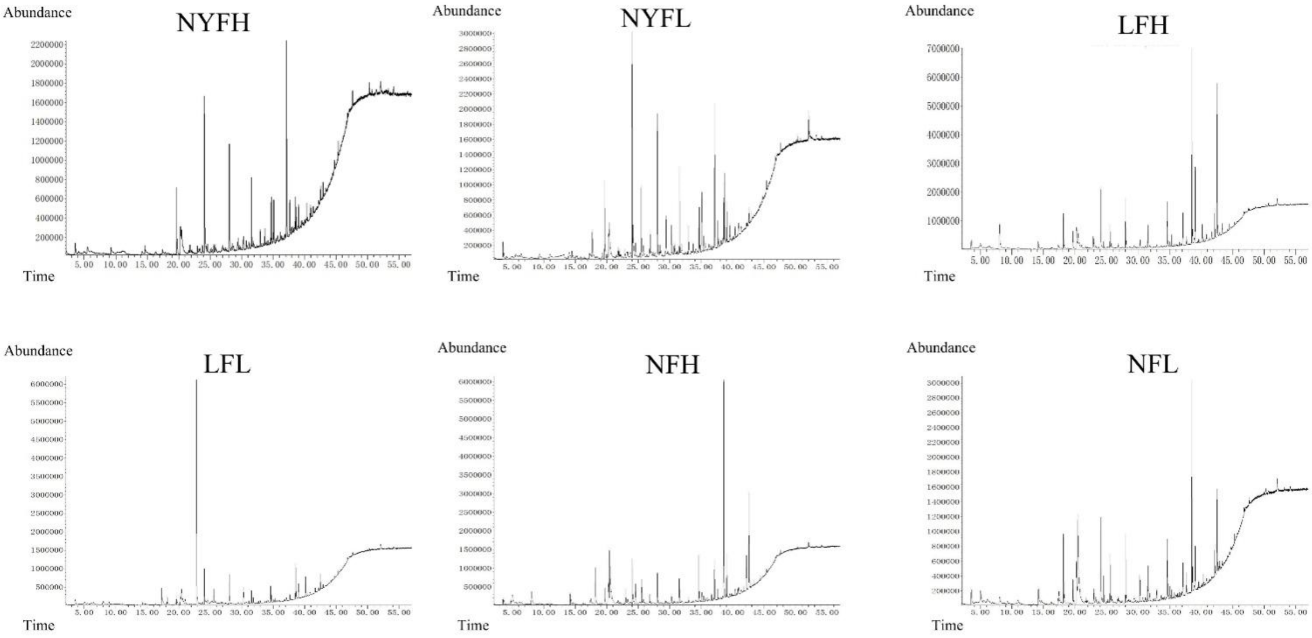


**Supplementary Material S2.**

| Sequence | Transducers | Responsive substances | Category substances |
|---|---|---|---|
| T1 | Sn_1 | Alkanes, Fumes | Propane, Natural gas, Fumes |
| T2 | Sn_2 | Alcohols, Aldehydes, Chain-breaking alkanes | Alcohol, Fumes, Isobutane, Formaldehyde |
| T3 | Sn_3 | Ozone (O3) | / |
| T4 | Sn_4 | Sulfide | Hydrogen sulfide |
| T5 | Sn_5 | Organic amine | Ammonia, Methylamine, Ethanolamine |
| T6 | Sn_6 | Organic gases, Phenyl ketones, Alcohols and aldehydes, Aromatic compounds | Toluene, Acetone, Ethanol, Hydrogen, Other organic vapours |
| T7 | Sn_7 | Short-chain alkanes | Methane, Natural gas, Biogas |
| T8 | Sn_8 | Aromatic compounds, Alcohols and aldehydes | Toluene, Formaldehyde, Benzene, Alcohol, Acetone |
| T9 | Sn_9 | Hydrogen-containing gas | Hydrogen (gas) |
| T10 | Sn_10 | Flammable gases | Methane CH4 |

Note: "/" indicates that the corresponding value was not found or could not be calculated.

## Supplementary Material S3.

| Form | Sequence | Compound name | CAS | Retention time/min | Relative content /μg/100g | | | | | |
|---|---|---|---|---|---|---|---|---|---|---|
| | | | | | NYXL（54） | NYXH（54） | NFL（52） | NFH（53） | LFL（56） | LFH（61） |
| Aldehydes | A1 | Acetaldeyde | 000075-07-0 | 4.10 | 3.71±0.02$^{a}$ | / | / | / | / | 2.61±0.03$^{b}$ |
| | A2 | Isovaleraldehyde | 000590-86-3 | 6.44 | 7.09±0.03$^{c}$ | / | / | 9.71±0.04$^{b}$ | / | 19.59±0.15$^{a}$ |
| | A3 | Hexanal | 000066-25-1 | 10.96 | 6.39±0.04$^{c}$ | 5.54±0.04$^{d}$ | 11.05±0.03a | / | 8.13±0.03$^{b}$ | / |
| | A4 | n-Heptanal | 000111-71-7 | 14.02 | 13.38±0.03$^{a}$ | / | / | / | / | / |
| | A5 | n-Octanal | 000124-13-0 | 17.25 | 14.30±0.03$^{e}$ | / | 34.23±0.19$^{b}$ | 29.07±0.06$^{c}$ | 35.68±0.06$^{a}$ | 16.70±0.11$^{d}$ |
| | A6 | n-Nonanal | 000124-19-6 | 20.28 | 20.19±0.03$^{f}$ | 173.62±0.05$^{d}$ | 250.31±0.09$^{b}$ | 271.36±0.06$^{a}$ | 195.68±0.23$^{c}$ | 117.50±0.19$^{e}$ |
| | A7 | trans-2-Nonenal | 018829-56-6 | 24.23 | 29.11±0.05$^{b}$ | 26.43±0.04$^{c}$ | 10.37±0.12$^{d}$ | 9.74±0.16$^{e}$ | 29.85±0.24$^{a}$ | / |
| | A8 | trans-2-decenal | 003913-81-3 | 26.88 | 26.59±0.06$^{a}$ | / | / | / | / | / |
| | A9 | 4-Ethylbenzaldehyde | 004748-78-1 | 28.50 | 12.34±0.06$^{b}$ | 17.21±0.04$^{a}$ | 6.39±0.14$^{c}$ | 3.92±0.08$^{d}$ | / | / |
| | A10 | Undecenoic aldehyde | 001337-83-3 | 29.41 | 32.13±0.08$^{a}$ | 26.69±0.05$^{b}$ | 8.79±0.07$^{d}$ | / | 11.36±0.06$^{c}$ | / |
| | A11 | Tridecanal | 010486-19-8 | 30.73 | 19.69±0.05$^{b}$ | 25.70±0.02$^{a}$ | 8.72±0.05$^{d}$ | 12.28±0.03$^{c}$ | / | / |
| | A12 | Myristaldehyde | 000124-25-4 | 32.96 | 29.85±0.07$^{b}$ | 42.82±0.03$^{a}$ | 18.01±0.04$^{d}$ | 19.09±0.04$^{c}$ | 15.73±0.08$^{e}$ | 9.43±0.08$^{f}$ |
| | A13 | Benzaldehyde, pentyl- | 006853-57-2 | 34.80 | 6.37±0.04$^{b}$ | 11.84±0.07$^{a}$ | / | / | / | / |
| | A14 | Pentadecanal | 002765-11-9 | 35.08 | 57.71±0.07$^{b}$ | 95.63±0.06$^{a}$ | 20.77±0.10$^{d}$ | 29.33±0.10$^{c}$ | 18.54±0.38$^{e}$ | 16.95±0.10$^{f}$ |
| | A15 | Hexadecanal | 000629-80-1 | 37.12 | 121.32±0.02$^{b}$ | 374.30±0.04$^{a}$ | 45.75±0.06$^{e}$ | 104.72±0.03$^{c}$ | 16.47±0.39$^{f}$ | 84.66±0.05d |
| | A16 | ctadecanal | 000638-66-4 | 40.93 | 13.15±0.02$^{c}$ | 32.16±0.05$^{a}$ | / | 15.53±0.21$^{b}$ | / | 9.52±0.05$^{d}$ |
| | A17 | 9-Octadecenal, (Z)- | 002423-10-1 | 41.36 | 7.23±0.05$^{b}$ | 25.68±0.11$^{a}$ | / | / | / | / |
| | A18 | [1,1'-Biphenyl]-4-carboxaldehyde | 003218-36-8 | 46.81 | 7.41±0.04$^{d}$ | 22.13±0.03$^{a}$ | 8.12±0.04$^{c}$ | 6.48±0.02$^{f}$ | 10.41±0.22$^{b}$ | 6.8±0.07$^{e}$ |
| | | **Subtotal** | | | 427.95±0.10$^{c}$ | 879.73±0.10$^{a}$ | 422.55±0.38$^{d}$ | 511.24±0.42$^{b}$ | 341.87±0.15$^{e}$ | 283.76±0.52$^{f}$ |
| Esters | B1 | Methyl caproate | 000106-70-7 | 14.02 | 5.13±0.04$^{f}$ | 12.28±0.04$^{e}$ | 50.28±0.03$^{b}$ | 58.59±0.04$^{a}$ | 28.31±0.10$^{d}$ | 34.61±0.09$^{c}$ |
| | B2 | Methyl octanoate | 000111-11-5 | 20.06 | 8.59±0.04$^{a}$ | / | / | / | / | / |
| | B3 | Methyl nonanoate | 001731-84-6 | 22.85 | 10.20±0.07$^{e}$ | 19.42±0.14$^{c}$ | 18.04±0.09$^{d}$ | 39.29±0.11$^{a}$ | / | 25.69±0.07$^{b}$ |

| | | | | | | | | | |
|---|---|---|---|---|---|---|---|---|---|
| B4 | Methyl decanoate | 000110-42-9 | 25.47 | 55.86±0.10$^{d}$ | 20.14±0.10$^{e}$ | 56.51±0.31$^{c}$ | 61.17±0.22$^{b}$ | 65.83±0.07$^{a}$ | 56.38±0.05$^{c}$ |
| B5 | gamma-Butyrolactone | 000096-48-0 | 26.76 | 7.93±0.04$^{b}$ | 6.78±0.07c | 4.58±0.04$^{f}$ | 6.33±0.11$^{d}$ | 9.18±0.05$^{a}$ | 5.49±0.02$^{e}$ |
| B6 | Methyl dodecanoate | 000111-82-0 | 30.31 | 7.55±0.06$^{e}$ | / | 24.33±0.05$^{d}$ | 35.34±0.07$^{a}$ | 29.55±0.42$^{b}$ | 27.18±0.07$^{c}$ |
| B7 | Methyl myristate | 000124-10-7 | 34.62 | 16.28±0.04$^{e}$ | 9.75±0.10$^{f}$ | 58.70±0.03$^{c}$ | 90.90±0.07$^{a}$ | 57.77±0.08$^{d}$ | 84.96±0.08$^{b}$ |
| B8 | Methyl myristoleate | 056219-06-8 | 35.37 | 10.33±0.05$^{c}$ | / | 9.28±0.03$^{d}$ | 10.41±0.03$^{c}$ | 18.12±0.09$^{b}$ | 23.07±0.06$^{a}$ |
| B9 | Methyl 12-methyltetradecanoate | 062691-05-8 | 35.70 | 13.77±0.08$^{a}$ | / | 8.82±0.08$^{d}$ | 9.32±0.10$^{c}$ | 11.09±0.05$^{b}$ | 8.09±0.05$^{e}$ |
| B10 | Methyl palmitate | 000112-39-0 | 38.56 | 30.39±0.16$^{f}$ | 52.69±0.04$^{e}$ | 211.66±0.29$^{c}$ | 427.64±0.09$^{a}$ | 127.08±0.04$^{d}$ | 383.08±0.06$^{b}$ |
| B11 | Hexamethylene diacrylate | 013048-33-4 | 38.67 | 47.09±0.07$^{a}$ | 24.69±0.03$^{d}$ | 24.76±0.10$^{d}$ | 22.24±0.06$^{e}$ | 35.30±0.20$^{b}$ | 33.09±0.02$^{c}$ |
| B12 | 7-Hexadecenoic acid, methyl ester, (Z)- | 056875-67-3 | 38.94 | 4.18±0.07$^{f}$ | 12.76±0.05$^{b}$ | 4.79±0.15$^{e}$ | 9.61±0.03$^{c}$ | 7.30±0.11$^{d}$ | 15.13±0.07$^{a}$ |
| B13 | methyl palmitoleate | 001120-25-8 | 39.06 | 32.17±0.05$^{f}$ | 47.81±0.11$^{d}$ | 46.58±0.03$^{e}$ | 88.0±0.05$^{b}$ | 56.38±0.29$^{c}$ | 150.56±0.07$^{a}$ |
| B14 | Diethyleneglycol dimethacrylate | 002358-84-1 | 39.59 | 9.73±0.08$^{c}$ | 9.84±0.09$^{c}$ | 11.06±0.16$^{a}$ | / | 8.69±0.05$^{d}$ | 10.85±0.03$^{b}$ |
| B15 | Methyl stearate | 000112-61-8 | 42.19 | 3.04±0.08$^{f}$ | 10.70±0.03$^{e}$ | 32.53±0.06$^{c}$ | 72.60±0.03$^{a}$ | 18.02±0.10$^{d}$ | 60.86±0.09$^{b}$ |
| B16 | Methyl elaidate C18:1T,trans-9 | 001937-62-8 | 42.55 | 12.02±0.04$^{f}$ | 35.80±0.13$^{e}$ | 84.84±0.18$^{c}$ | 189.25±0.10$^{b}$ | 58.18±0.04$^{d}$ | 300.09±0.71$^{a}$ |
| B17 | Methyl linoleate | 000112-63-0 | 43.35 | 4.38±0.07$^{f}$ | 10.62±0.10$^{c}$ | 6.81±0.07$^{d}$ | 13.32±0.11$^{b}$ | 5.48±0.13$^{e}$ | 23.03±0.12$^{a}$ |
| B18 | Butyl phthalate | 000084-74-2 | 44.22 | 2.28±0.03$^{e}$ | 16.35±0.07$^{a}$ | 3.80±0.02$^{d}$ | / | 7.47±0.06$^{b}$ | 4.44±0.07c |
| B19 | Methyl acetate | 000079-20-9 | 5.00 | / | 17.80±0.19$^{e}$ | 40.42±0.09$^{b}$ | 65.09±0.02$^{a}$ | 33.16±0.04$^{c}$ | 32.35±0.17$^{d}$ |
| B20 | Methyl heptanoate | 000106-73-0 | 17.29 | / | 5.67±0.05$^{c}$ | 9.08±0.03$^{b}$ | 11.64±0.07$^{a}$ | / | / |
| B21 | Methyl hydrocinnamate | 000103-25-3 | 31.35 | / | 2.74±0.06$^{a}$ | 1.7±0.05$^{b}$ | / | / | / |
| B22 | gamma-decalactone | 000706-14-9 | 35.37 | / | 5.97±0.17$^{a}$ | / | / | / | / |
| B23 | Methyl pentadecanoate | 007132-64-1 | 36.64 | / | 3.69±0.03$^{e}$ | 7.46±0.06$^{c}$ | 12.75±0.07$^{a}$ | 5.83±0.05$^{d}$ | 10.31±0.04$^{b}$ |

| | | | | | | | | | | |
|---|---|---|---|---|---|---|---|---|---|---|
| | B24 | Methyl butyrate | 000623-42-7 | 8.06 | / | / | 22.83±0.083$^{c}$ | / | 47.69±0.27$^{b}$ | 134.58±0.06$^{a}$ |
| | B25 | Methyl isobutyrate | 000547-63-7 | 6.51 | / | / | / | / | 21.06±0.07$^{a}$ | / |
| | B26 | Methyl 2-methylbutyrate | 000868-57-5 | 8.65 | / | / | / | / | 8.17±0.04$^{a}$ | 1.58±0.04$^{b}$ |
| | B27 | Propanoic acid, 2-hydroxy-, methyl ester | 000547-64-8 | 18.18 | / | / | 95.73±0.09$^{c}$ | 103.63±0.04a | 45.38±0.03$^{d}$ | 101.41±0.04$^{b}$ |
| | B28 | Methyl cis-10-heptadecenoate C17:1,cis-10 | 075190-82-8 | 40.83 | / | / | 10.36±0.05$^{b}$ | / | / | 22.91±0.28$^{a}$ |
| | B29 | Methyl oleate | 000112-62-9 | 42.65 | / | / | 13.11±0.09$^{c}$ | 26.01±0.62$^{b}$ | / | 29.68±0.65$^{a}$ |
| | B30 | ETHYL STEARATE | 000111-61-5 | 42.79 | / | / | 5.14±0.05$^{a}$ | / | 1.56±0.10$^{b}$ | / |
| | B31 | Methyl 13-methyltetradecanoate | 1000424-50-7 | 36.00 | / | / | / | 10.37±0.06$^{a}$ | / | 5.25±0.06$^{b}$ |
| | B32 | Methyl heptadecanoate | 001731-92-6 | 39.81 | / | / | / | 6.86±0.04$^{b}$ | / | 14.36±0.06$^{a}$ |
| | B33 | ETHYL LINOLEOLATE | 000544-35-4 | 44.43 | / | / | / | 6.44±0.05$^{b}$ | / | 10.41±0.05$^{a}$ |
| | B34 | Benzyl benzoate | 000120-51-4 | 45.71 | / | / | / | 2.87±0.13$^{a}$ | / | / |
| | B35 | Methyl isovalerate | 000556-24-1 | 8.96 | / | / | / | / | 22.11±0.11$^{a}$ | / |
| | B36 | Methyl phthalate | 000131-11-3 | 40.16 | / | / | / | / | 64.21±0.31$^{a}$ | / |
| | B37 | Methyl valerate | 000624-24-8 | 10.98 | 10.70±24.62$^{a}$ | / | / | / | / | / |
| | | **Subtotal** | | | 280.91±0.43$^{f}$ | 325.49±0.49$^{e}$ | 863.22±0.75$^{c}$ | 1379.74±0.81$^{b}$ | 792.91±1.31$^{d}$ | 1617.02±0.96$^{a}$ |
| **Alcohols** | C1 | Isopentanol | 000123-51-3 | 15.25 | 3.39±0.10$^{a}$ | / | / | / | / | / |
| | C2 | n-Hexanol | 000111-27-3 | 19.25 | 8.32±0.06$^{a}$ | / | 3.89±0.03$^{c}$ | 4.70±0.06$^{b}$ | / | / |
| | C3 | 1-Octen-3-ol | 003391-86-4 | 21.77 | 10.15±0.23$^{c}$ | 18.58±0.03$^{a}$ | 6.9±0.16$^{e}$ | 10.77±0.05$^{b}$ | / | 8.06±0.08$^{d}$ |
| | C4 | N-OCTANOL | 000111-87-5 | 24.55 | 17.48±0.03$^{e}$ | 28.88±0.11$^{d}$ | 37.65±0.06$^{c}$ | 51.07±0.06$^{a}$ | 38.51±0.1$^{b}$ | 15.44±0.07$^{f}$ |
| | C5 | n-Dodecanol | 000112-53-8 | 33.69 | 6.27±0.05$^{c}$ | 28.64±0.24$^{a}$ | 5.55±0.14$^{e}$ | 5.86±0.07$^{d}$ | 12.30±0.08$^{b}$ | 5.63±0.04$^{e}$ |

| | | | | | | | | | |
|---|---|---|---|---|---|---|---|---|---|
| | C6 | [1,1'-Biphenyl]-4-methanol | 003597-91-9 | 53.25 | 6.9±0.07$^{b}$ | 14.84±0.09$^{a}$ | 3.13±0.04$^{c}$ | / | / | / |
| | C7 | Phenethyl alcohol | 000060-12-8 | 32.70 | / | 2.51±0.04$^{a}$ | / | / | / | / |
| | C8 | Benzyl alcohol | 000100-51-6 | 31.93 | / | / | / | / | / | 5.19±0.07$^{a}$ |
| | | **Subtotal** | | | 52.53±0.31$^{d}$ | 93.45±0.17$^{a}$ | 57.11±0.34$^{c}$ | 72.39±0.02$^{b}$ | 50.81±0.17$^{e}$ | 34.32±0.15$^{f}$ |
| | D1 | 3-Hydroxy-2-butanone | 000513-86-0 | 17.71 | 36.6±0.30$^{b}$ | 29.30±0.03$^{c}$ | / | / | 104.85±0.07$^{a}$ | 14.76±0.09$^{d}$ |
| **Ketones** | D2 | cis-Geranylacetone | 003879-26-3 | 31.50 | 1.83±0.09$^{e}$ | 6.09±0.04$^{d}$ | 14.54±0.24$^{b}$ | 45.28±0.09$^{a}$ | 6.19±0.04$^{d}$ | 11.91±0.04$^{c}$ |
| | D3 | 5-Hepten-2-one, 6-methyl- | 000110-93-0 | 18.86 | / | / | / | 4.22±0.01$^{b}$ | 13.09±0.30$^{a}$ | / |
| | | **Subtotal** | | | 38.43±0.39$^{c}$ | 35.38±0.05$^{d}$ | 14.54±0.24$^{f}$ | 49.50±0.10$^{b}$ | 124.13±0.33$^{a}$ | 26.67±0.12$^{e}$ |
| | E1 | Pentadecane | 000629-62-9 | 23.29 | 7.91±0.10$^{f}$ | 21.33±0.04$^{c}$ | 15.50±0.02$^{d}$ | 24.51±0.03$^{a}$ | 22.92±0.10$^{b}$ | 12.09±0.06$^{e}$ |
| | E2 | Hexeadecane | 000544-76-3 | 25.79 | 14.70±0.03$^{e}$ | 23.02±0.03$^{b}$ | 13.90±0.04$^{f}$ | 19.24±0.05$^{c}$ | 25.24±0.03$^{a}$ | 16.17±0.05$^{d}$ |
| | E3 | Dodecane, 4,6-dimethyl- | 061141-72-8 | 16.37 | / | 13.14±0.16a | / | / | / | 5.53±0.05$^{b}$ |
| | E4 | Oxirane, tetradecyl- | 007320-37-8 | 36.18 | / | 21.56±0.07$^{a}$ | / | / | / | / |
| | E5 | N-OCTANE n-Tetradecane | 000111-65-9 | 4.83 | / | / | 4.89±0.10$^{c}$ | 22.24±0.08$^{a}$ | / | 12.20±0.07$^{b}$ |
| | E6 | n-Tetradecane | 000629-59-4 | 20.70 | / | / | 68.65±0.06$^{a}$ | 53.62±0.08$^{b}$ | / | 36.11±0.07$^{c}$ |
| **Hydrocarbons** | E7 | (-)-ARISTOLENE | 006831-16-9 | 28.63 | 4.76±0.06$^{b}$ | 10.20±0.06a | / | / | / | 2.28±0.03$^{c}$ |
| | E8 | α-curcumene | 000644-30-4 | 29.67 | 5.61±0.03$^{b}$ | 14.81±0.02$^{a}$ | / | 4.95±0.06$^{c}$ | / | 4.07±0.06$^{d}$ |
| | E9 | 2-Hexadecene, 2,6,10,14-tetramethyl- | 056554-34-8 | 30.27 | 22.53±0.07$^{d}$ | 39.03±0.54$^{b}$ | 24.85±0.08$^{c}$ | / | 58.96±0.57$^{a}$ | / |
| | E10 | 3,7,11,15-Tetramethylhexadec-2-ene | 002437-93-6 | 31.86 | 10.98±0.034$^{d}$ | 26.19±0.06$^{b}$ | 14.71±0.04$^{c}$ | 10.69±0.03$^{e}$ | 29.69±0.33$^{a}$ | 4.98±0.06$^{f}$ |
| | E11 | Cuparene | 016982-00-6 | 30.87 | / | 7.81±0.04$^{a}$ | / | / | 5.91±0.04$^{b}$ | 1.83±0.05$^{c}$ |
| | E12 | (+)-DELTA- | 000483-76-1 | 29.43 | / | / | / | 6.33±0.09$^{a}$ | / | / |

| | | | | | | | | | | |
|---|---|---|---|---|---|---|---|---|---|---|
| | | CADINENE | | | | | | | | |
| | | **Subtotal** | | | 66.48±0.05[e] | 177.09±0.47[a] | 142.49±0.20[b] | 141.58±0.16[c] | 142.72±0.21[b] | 95.27±0.16[d] |
| **Other categories** | F1 | Carbamodithioic acid, diethyl-, methyl ester | 000686-07-7 | 34.99 | / | 19.24±0.06[a] | / | / | / | / |
| | F2 | Naphthalene | 000091-20-3 | 29.32 | 3.75±0.21[c] | 20.20±0.76[a] | 5.82±0.04[c] | 9.67±0.05[b] | 5.55±0.11[c] | 9.25±0.06[b] |
| | F3 | 2-Acetylpyrrole | 001072-83-9 | 34.11 | 1.24±0.09[c] | / | / | 11.66±0.26[a] | / | 6.37±0.10[b] |
| | F4 | 4-Methylbiphenyl | 000644-08-6 | 36.84 | 3.07±0.03[d] | / | 5.14±0.08[c] | / | 6.47±0.04[a] | 5.67±0.05[b] |
| | F5 | Benzene, 1,2,3,5-tetramethyl- | 000527-53-7 | 21.64 | / | 11.06±0.07[a] | / | 6.68±0.07[c] | / | 7.33±0.11[b] |
| | F6 | Phenol | 000108-95-2 | 34.51 | / | 4.68±0.05[a] | / | / | 3.20±0.03[c] | 3.38±0.04[b] |
| | F7 | 2,4-Di-tert-butylphenol | 000096-76-4 | 40.00 | / | 9.42±0.05[a] | / | / | / | / |
| | F8 | Tetramethylpyrazine | 001124-11-4 | 22.85 | / | / | / | 6.09±0.05[c] | 937.85±0.05[a] | 30.22±0.53[b] |
| | F9 | 2,3-Dimethylpyrazine | 005910-89-4 | 19.46 | / | / | / | / | 19.54±0.10[a] | / |
| | F10 | 2,3,5-Trimethylpyrazine | 014667-55-1 | 21.04 | / | / | / | / | 51.46±0.09[a] | 19.44±0.06[b] |
| | F11 | 2-Ethyl-3,5-dimethylpyrazine | 013925-07-0 | 22.43 | / | / | / | / | 6.05±0.09[a] | / |
| | F12 | 2,3,5-Trimethyl-6-ethylpyrazine | 017398-16-2 | 23.76 | / | / | / | / | 17.85±0.11[a] | 8.68±0.18[b] |
| | F13 | p-Cresol | 000106-44-5 | 36.02 | / | 6.85±0.13[b] | / | / | 11.16±0.05[a] | / |
| | F14 | Thiophene, 2,5-dihydro- | 001708-32-3 | 41.69 | / | / | 3.59±0.06[d] | 12.52±0.05[c] | 19.62±0.07[a] | 14.53±0.16[b] |
| | F15 | Thiophene, 2,3-dihydro- | 001120-59-8 | 40.81 | / | / | / | / | 21.22±0.04a | / |
| | F16 | Dimethyl sulfone | 000067-71-0 | 32.69 | / | / | | / | 8.50±0.05a | 2.69±0.03b |
| | | **Subtotal** | | | 8.06±0.15[f] | 71.45±0.75[c] | 14.55±0.09[e] | 46.64±0.40[d] | 1108.47±0.30[a] | 102.67±7.87[b] |

| | | | | | | |
|---|---|---|---|---|---|---|
| **Total** | 874.36±0.66$^{f}$ | 1582.59±0.84$^{d}$ | 1514.46±0.83$^{e}$ | 2201.09±1.41$^{b}$ | 2560.90±2.13$^{a}$ | 2159.70±7.18$^{c}$ |

Note: "/" indicates that the corresponding value was not found or could not be calculated.
